# Supplementary material for: Identification of ETV6-RUNX1-like and DUX4-rearranged subtypes in paediatric B-cell precursor acute lymphoblastic leukaemia
Source: Nat Commun. 2016 Jun 6;7:11790. doi: 10.1038/ncomms11790 (PMC4897744; doi:10.1038/ncomms11790)
Supplement: Supplementary Information — Supplementary Figures 1-19, Supplementary Tables 1-2 and Supplementary References. [file ncomms11790-s1.pdf]

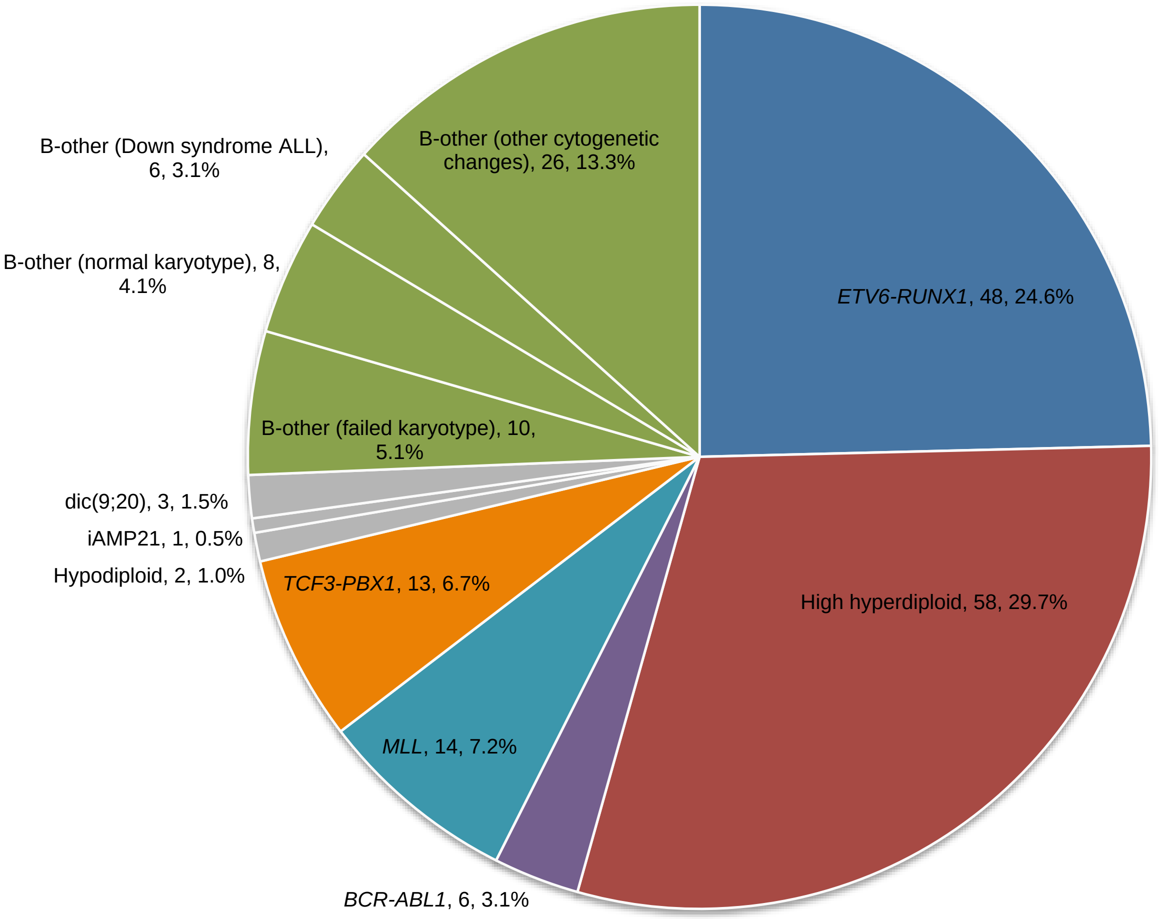

Supplementary Figure 1. Distribution of the 195 BCP-ALL cases among thirteen genetic subtypes.

The subtypes “High hyperdiploid” (51-67 chromosomes) and “Hypodiploid” (<45 chromosomes) excludes cases positive for *BCR-ABL1*, *ETV6-RUNX1*, *MLL*, *TCF3-PBX1* and iAMP21. Of the 50 cases classified as B-other, 41 were negative for *BCR-ABL1*, *ETV6-RUNX1*, *MLL*, *TCF3-PBX1* and iAMP21, while nine cases had incomplete testing for one or more of these aberrations. Three cases originally classified as B-other were reclassified as *ETV6-RUNX1* or *MLL*, based on RNA-seq findings.

Supplementary Figure 2. Identification of *IGH-DUX4* breakpoints.

Reads from RNA-sequencing (RNA-seq), whole exome sequencing (WES), and mate pair whole genome sequencing (MP-WGS) that support a *IGH-DUX4* rearrangements are visualized using IGV[1] for the cases (**a**) #35, (**b**) #47, (**c**) #53, (**d**) #67, (**e**) #124, (**f**) #174, and (**g**) #179. Within each IGV window, the sequencing reads are illustrated below the chromosomal coordinates and include RNA-seq (top), WES (middle), and MP-WGS (bottom). WES data for breakpoint detection was available for the cases #35, #47, #174, and #179. The *DUX4* coding region or genes within *IGH* are indicated below the sequencing reads. The detected breakpoints are indicated by a red line. The visualization only contains read pairs consisting of one read within *IGH* and one read in the *DUX4*, and thus supporting a breakpoint involving these regions. Reads in the RNA-seq and WES libraries will be directed towards the breakpoint they support whereas reads in the MP-WGS library will be directed away from the breakpoint. The insert sizes were between 1-8 kb for MP-WGS libraries and between 200-400 for RNA-seq and WES libraries. The exact 5' breakpoints for case #35 and the exact 3' breakpoints for case #67 could not be conclusively determined from the visualized reads. The exact breakpoints for these cases were instead detected by guided assembly of all RNA-seq reads mapping to the *DUX4* region, followed by alignment of "overhang" regions.

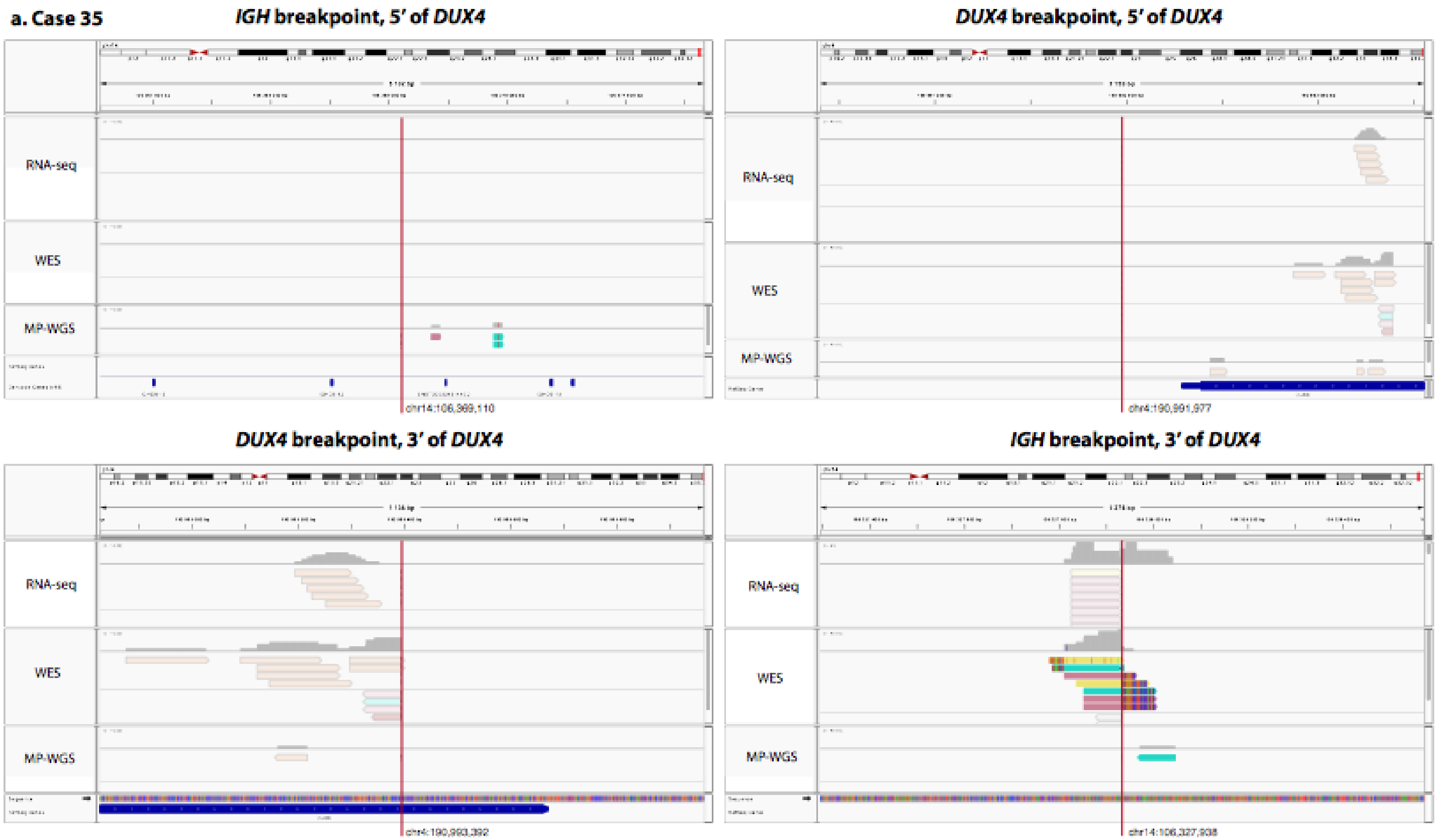

Supplementary Figure 2a.

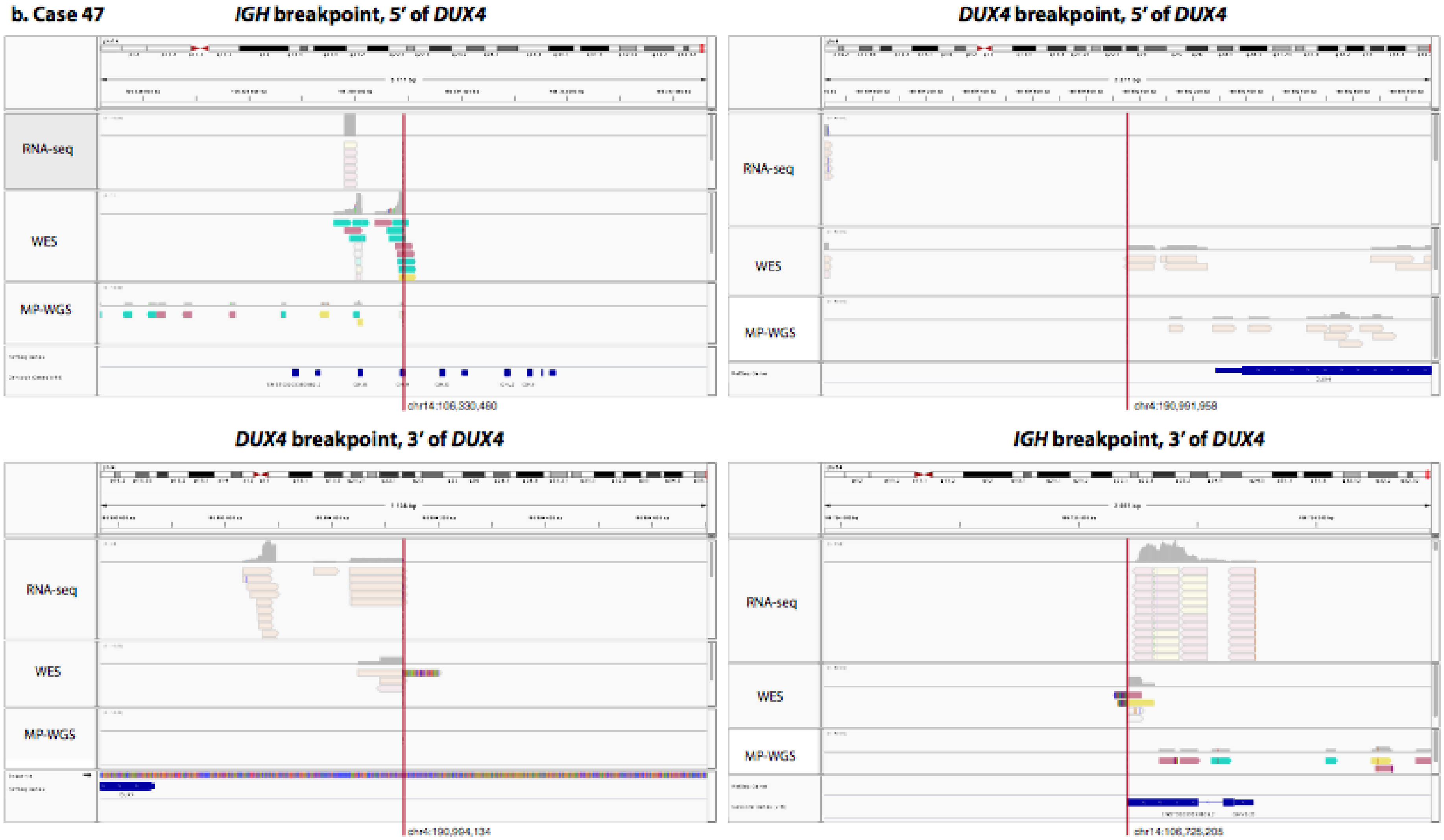

Supplementary Figure 2b.

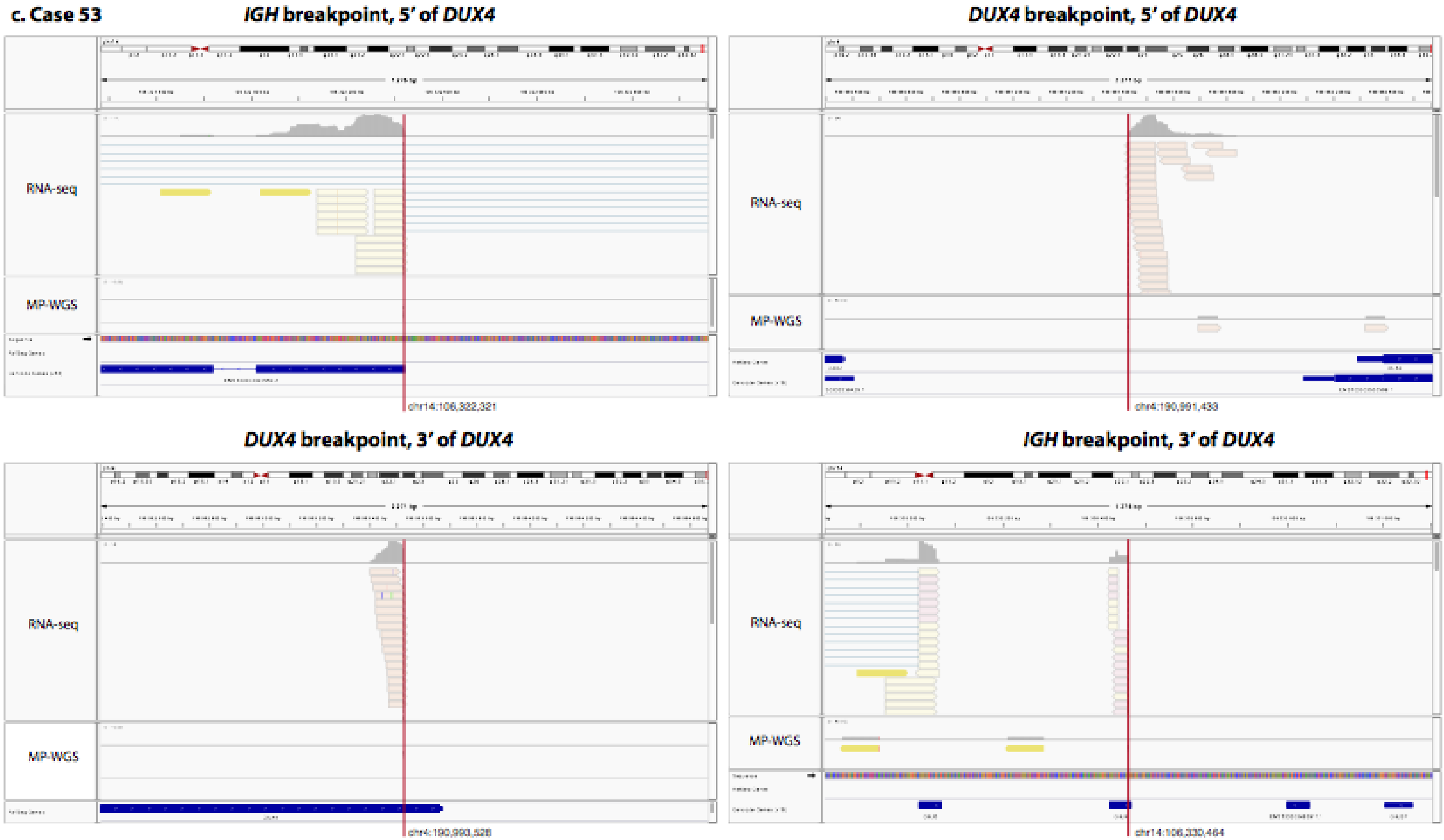

Supplementary Figure 2c.

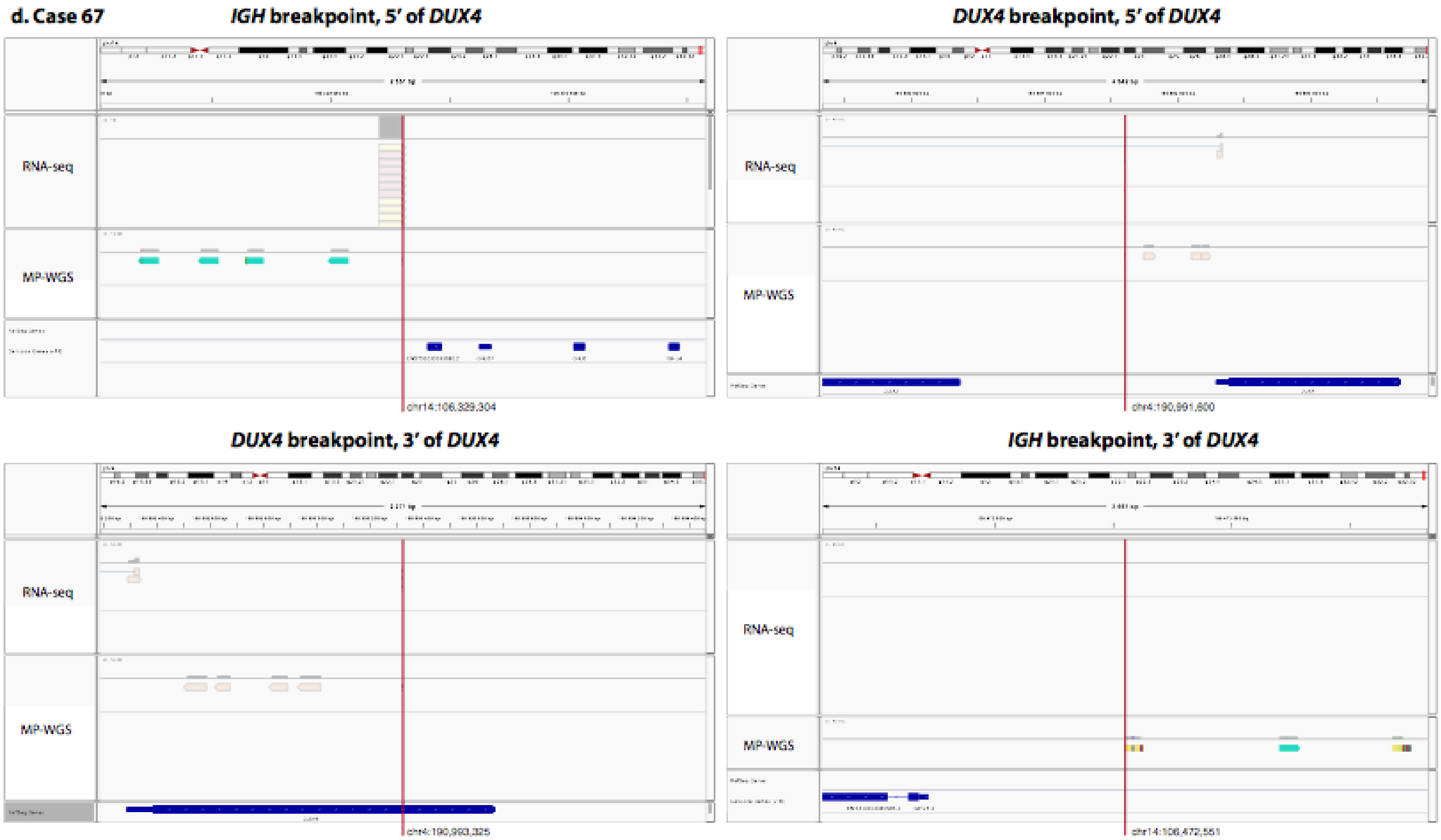

Supplementary Figure 2d.

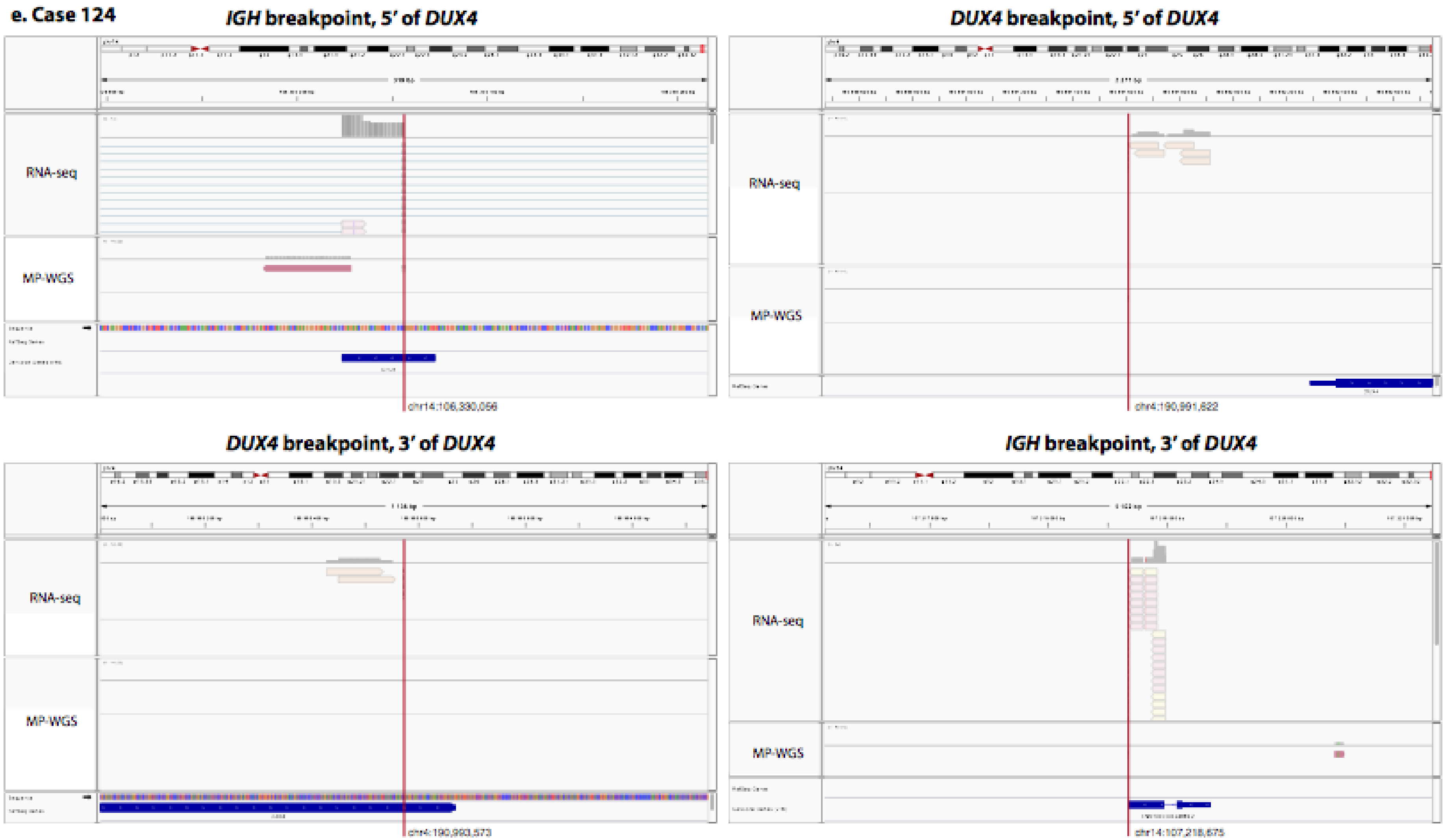

Supplementary Figure 2e.

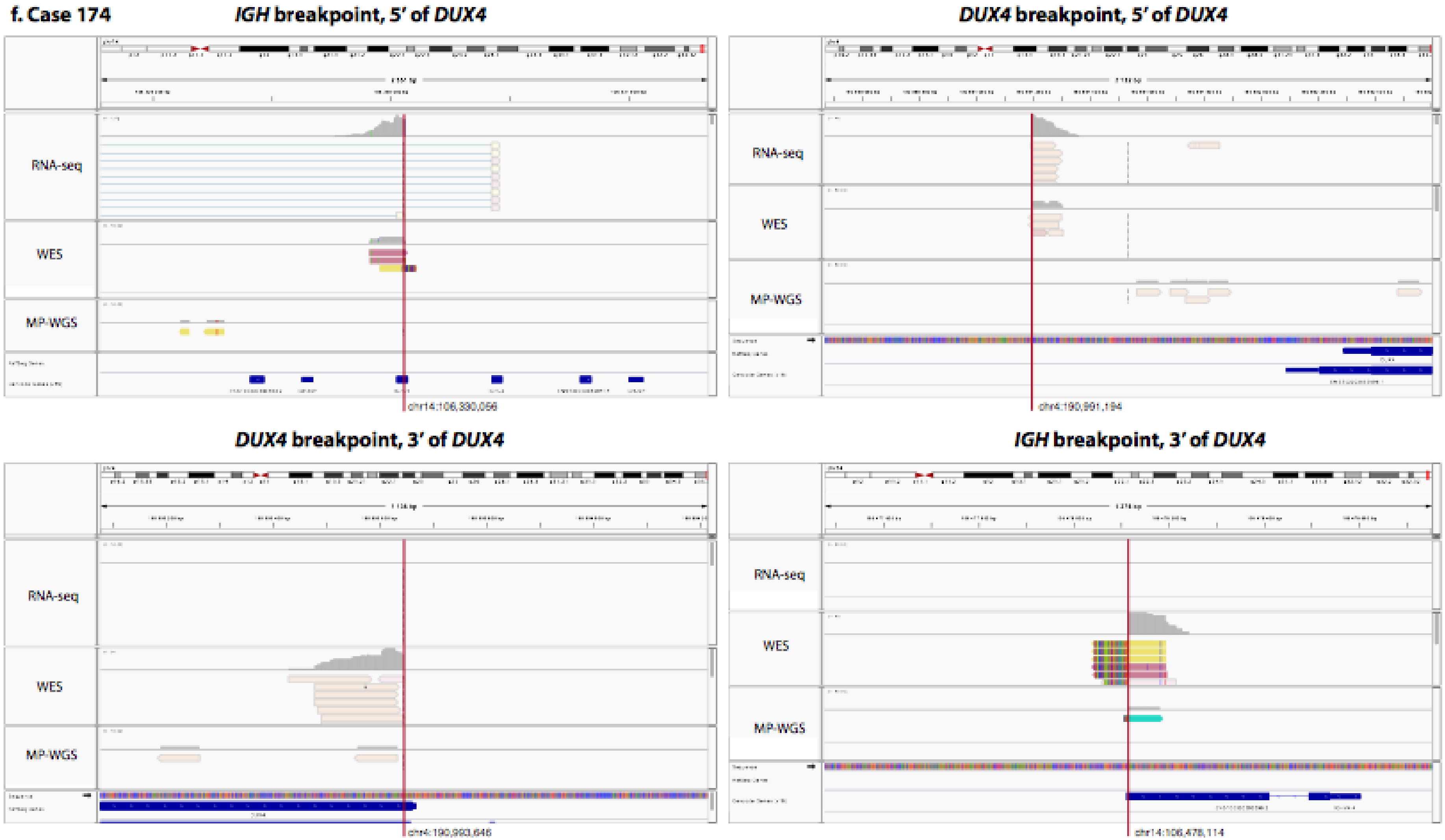

Supplementary Figure 2f.

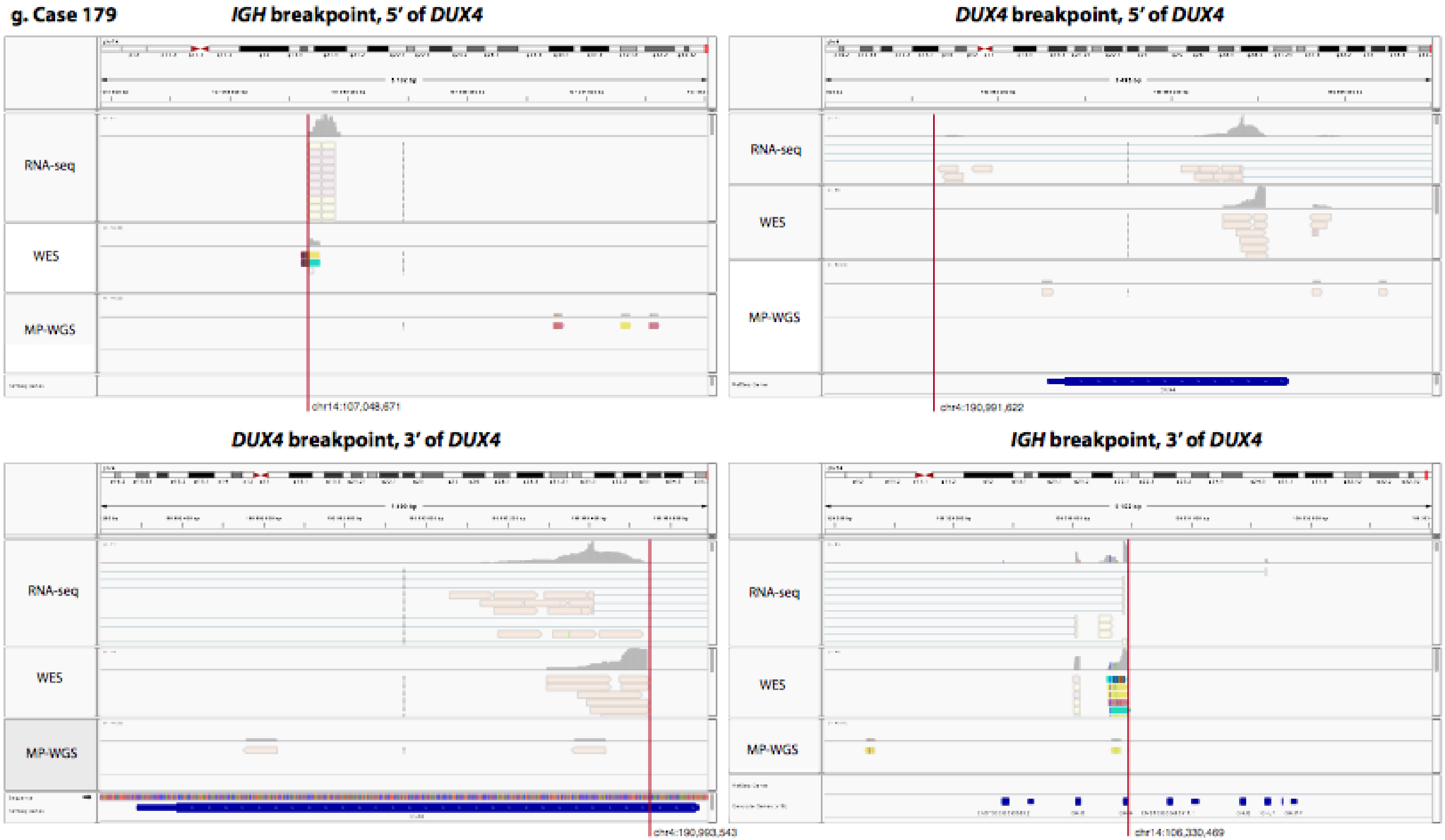

Supplementary Figure 2g.

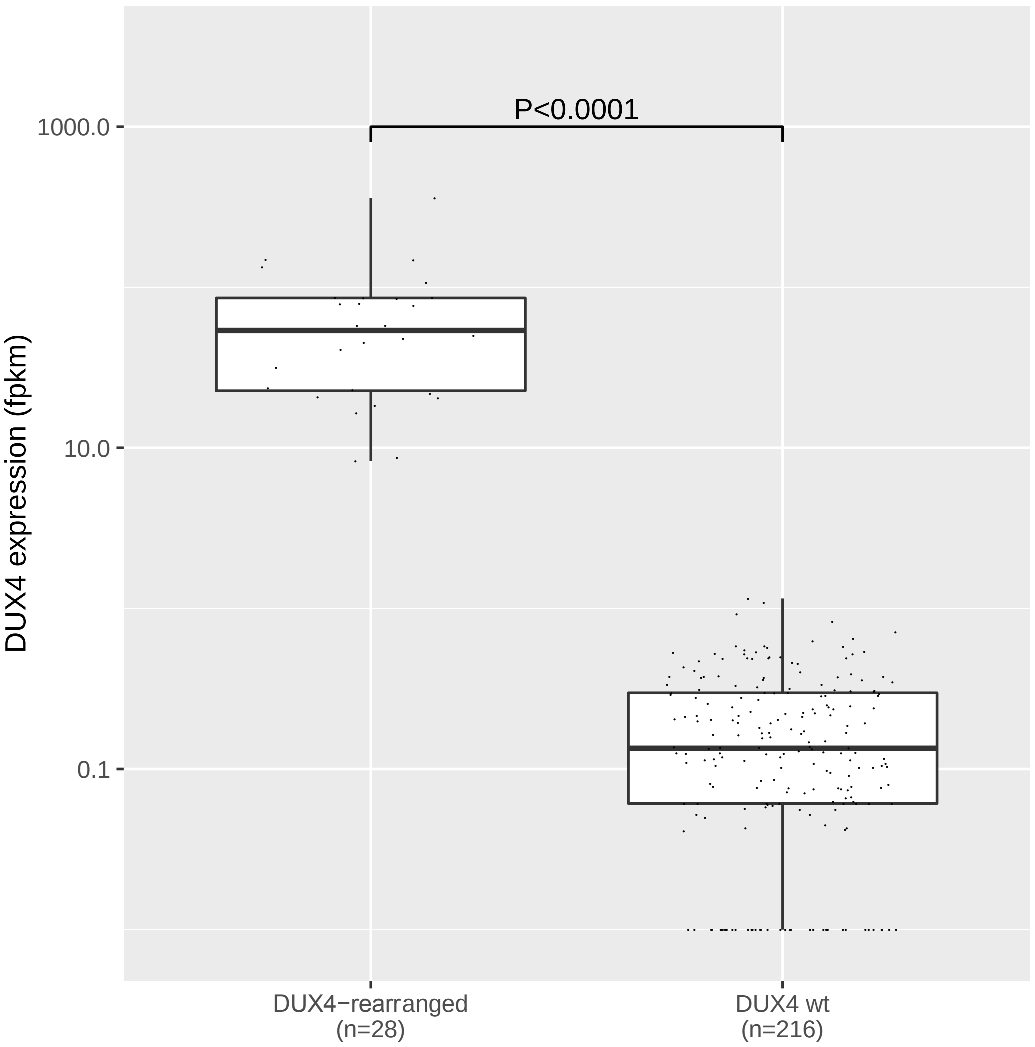

Supplementary Figure 3. *DUX4* expression in *DUX4*-rearranged cases.
DUX4 gene expression in 244 BCP ALL cases with and without *DUX4* rearrangement from the combined discovery and validation cohorts. *DUX4* is significantly overexpressed in *DUX4*-rearranged cases. The boxes are defined by the first and third quartiles and whiskers extend from the boxes to the highest and lowest values. Two sided *P*-value calculated using Mann-Whitney U test.

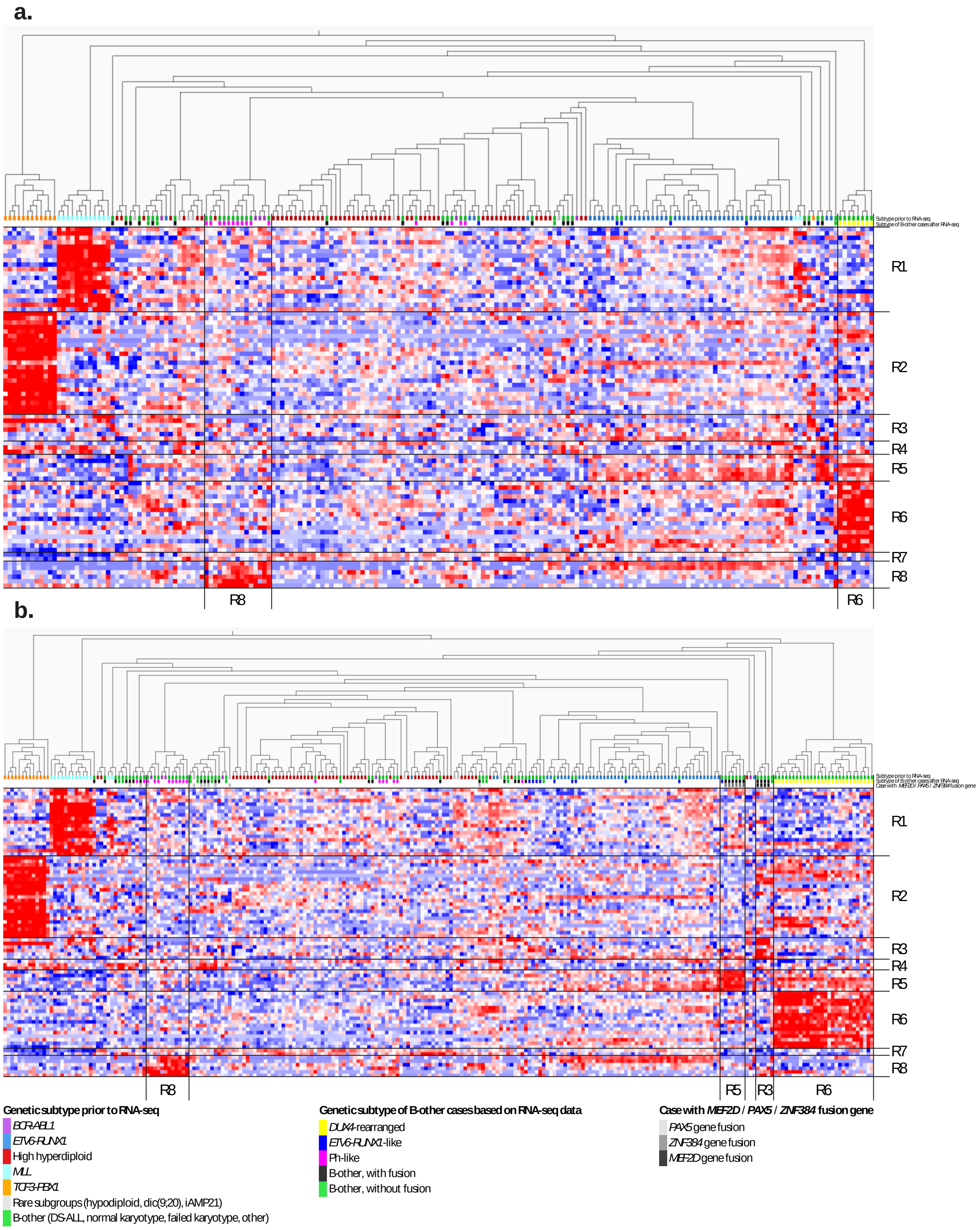

Supplementary Figure 4. Hierarchical clustering based on the eight "ROSE" gene sets (R1-R8) described by Harvey et al[2].

(**a**) Hierarchical clustering of the 195 BCP ALL cases in the discovery cohort. Three *BCR-ABL1*-positive cases and 11 cases from the B-other group form a cluster with high expression of genes from the R8 gene set, indicating a Ph-like expression profile. In addition, all eight cases with *DUX4* rearrangement cluster together and exhibit high expression of the genes from the R6 gene set. (**b**) Hierarchical clustering of the 244 BCP ALL cases in the combined discovery and validation cohorts. All 28 *DUX4* rearranged cases cluster together and exhibit high expression of the genes from the R6 gene set. In

addition, all six cases with *ZNF384* (*EP300-ZNF384*, n=4; *TAF15-ZNF384*, n=1, *TCF3-ZNF384*, n=1) cluster together and exhibit high expression of the genes of the R5 gene set. All four cases with fusions involving *MEF2D* (*MEF2D-HNRNPUL1*, n=3; *MEF2D-FOXJ2*, n=1) cluster together and exhibit high expression of the genes of the R3 gene set.

a.

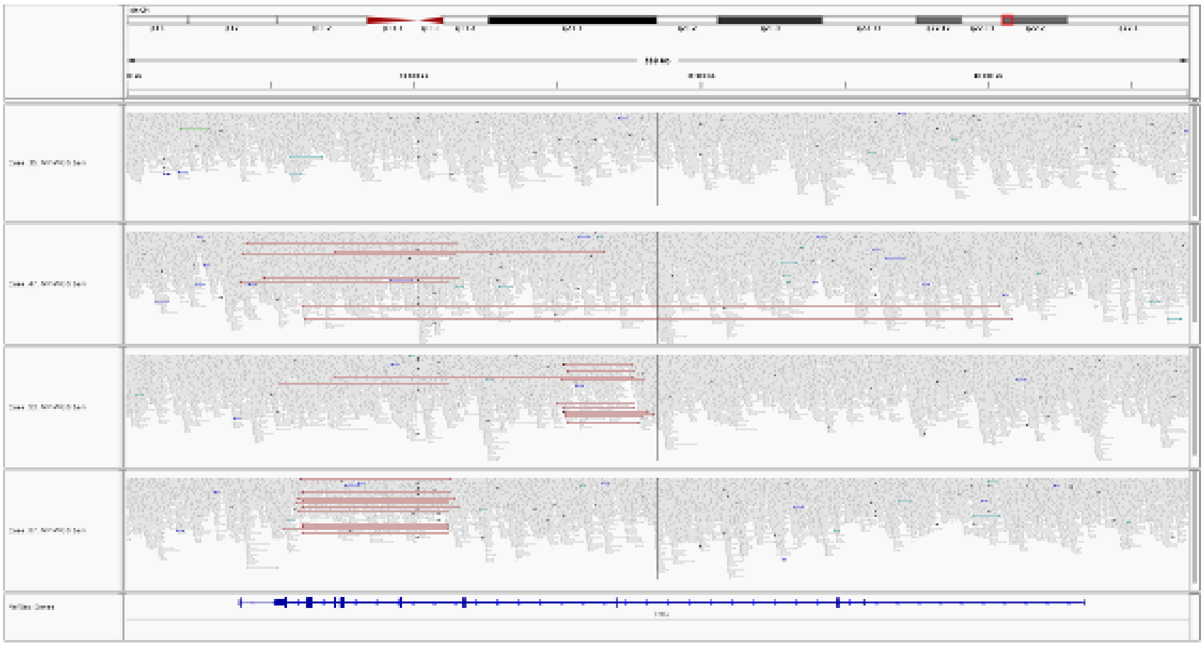

b.

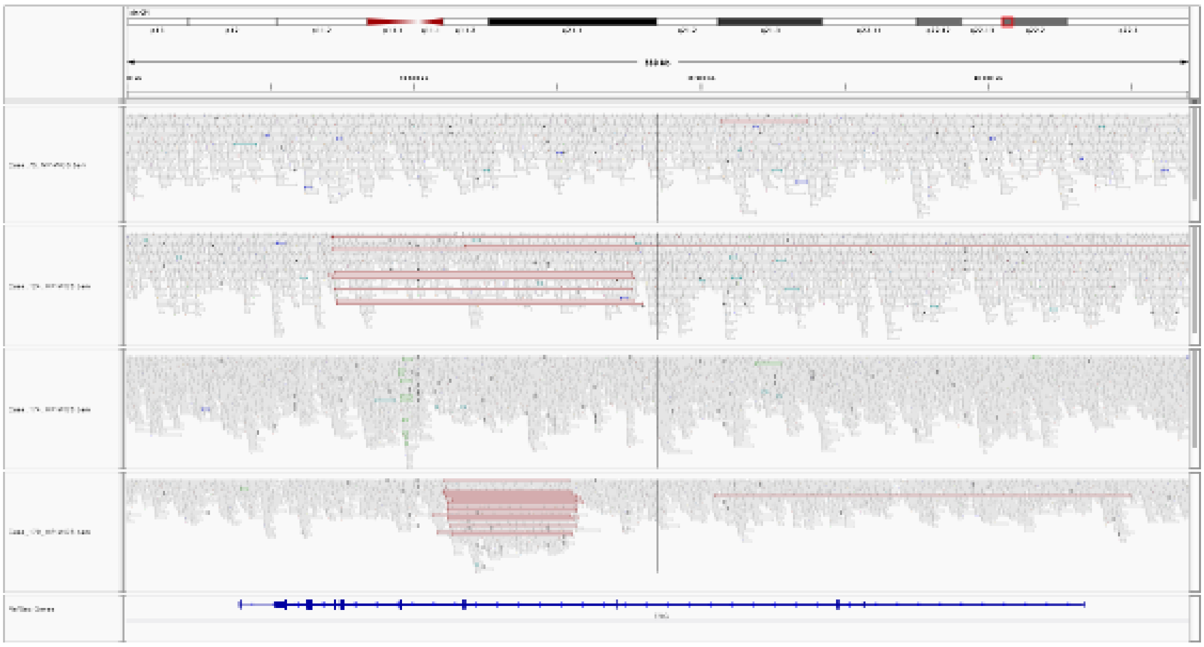

Supplementary Figure 5. *ERG* deletions in *DUX4*-rearranged cases.
ERG deletions in *DUX4*-rearranged cases detected by mate pair whole genome sequencing (MP-WGS) and visualized using IGV[1]. (**a**) cases #35, #47, #53, and #67, and (**b**) cases #75, #124, #174, and #179. The insert sizes for MP-WGS were 1-8 kb. Read pairs mapped > 20 kb apart, indicating a deletion, are indicated in red. Read pairs indicating intragenic *ERG* deletions were detected in cases #47, #53, #67, #124, and #179.

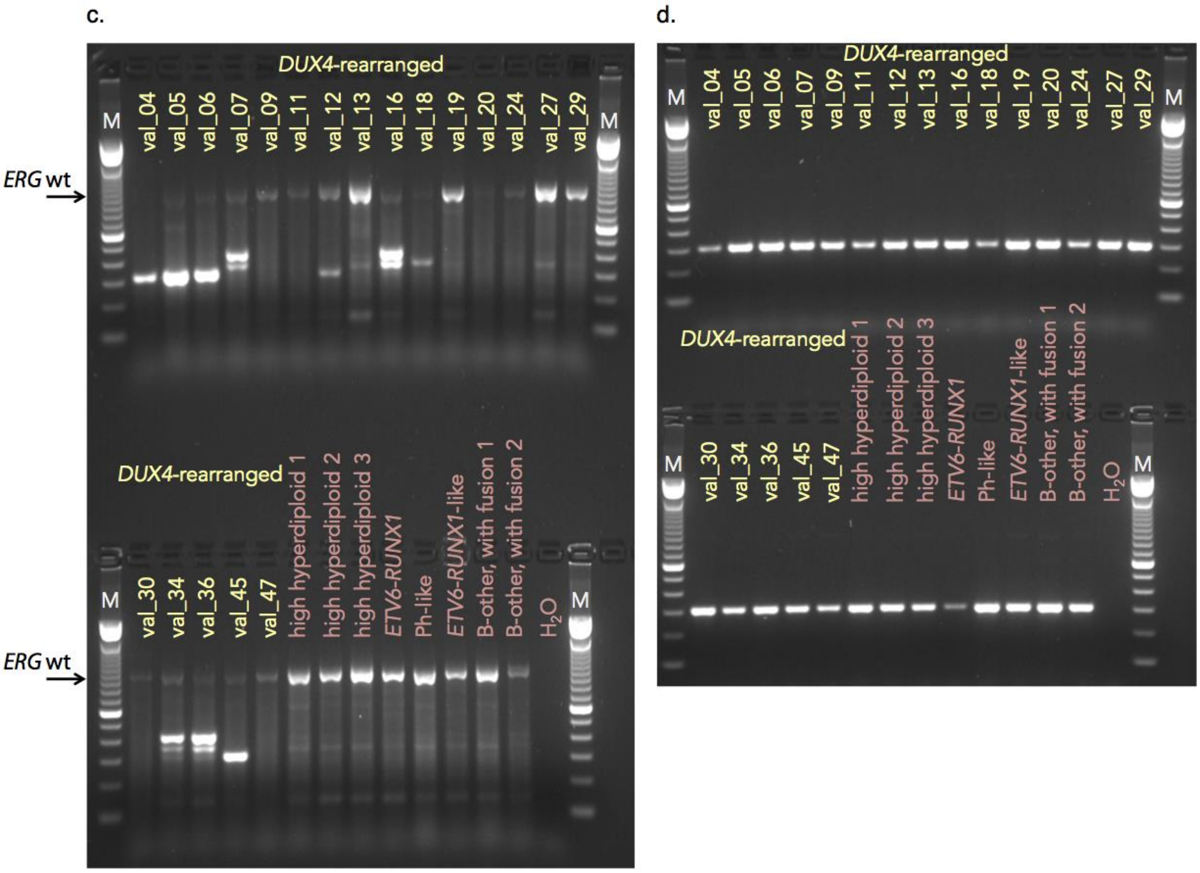

Supplementary Figure 5c-d.
ERG deletions in *DUX4*-rearranged cases in the validation cohort detected indirectly by RT-PCR. (**c**) RT-PCR of 20 BCP ALL cases with and eight BCP ALL cases without *DUX4* rearrangement. 10/20 cases with *DUX4* rearrangement express truncated *ERG* transcripts, indicating *ERG* deletions. (**d**) RT-PCR for *ABL1* verifying integrity of cDNA for all cases.

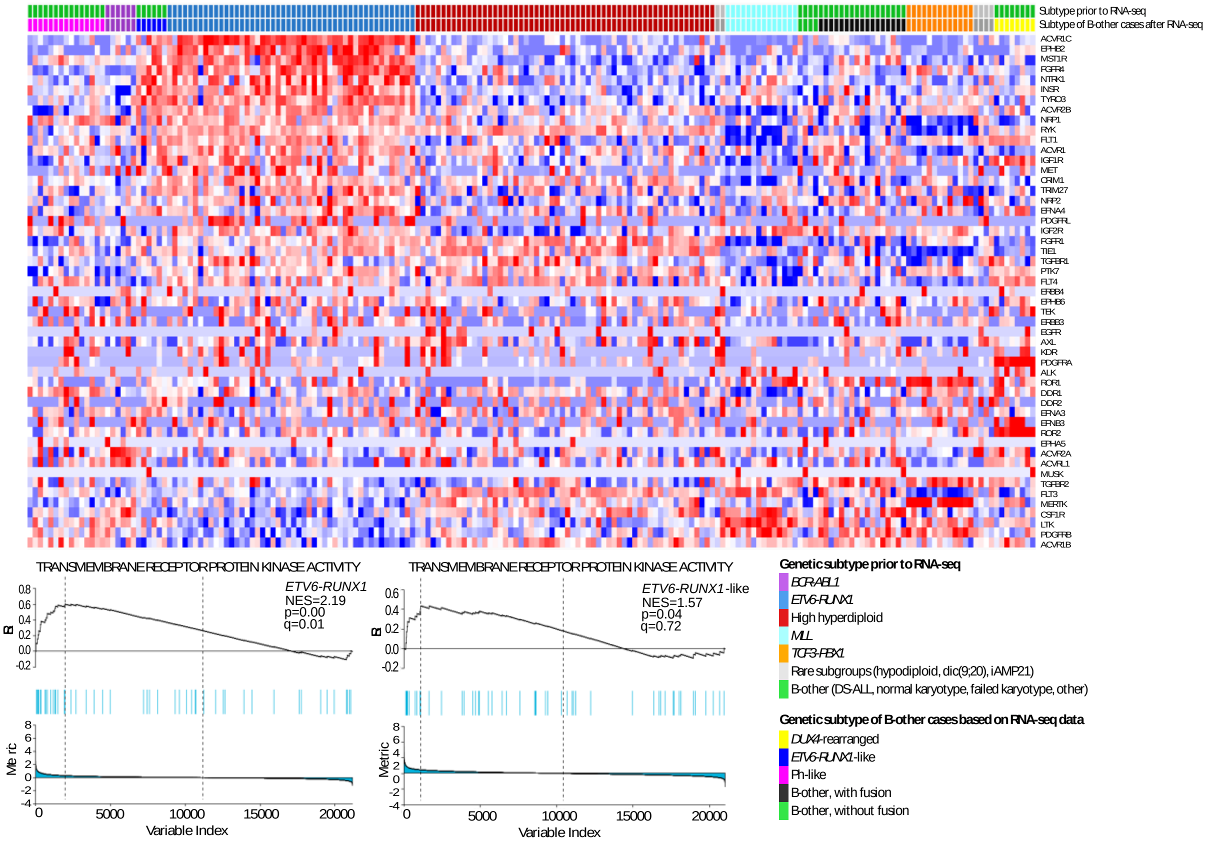

Supplementary Figure 6. Expression of transmembrane receptor protein kinase activity genes in 195 BCP ALLs.
Expression profile of genes involved in transmembrane receptor protein kinase activity, highlighted by gene set enrichment analysis to be enriched in *ETV6-RUNX1*-positive cases.

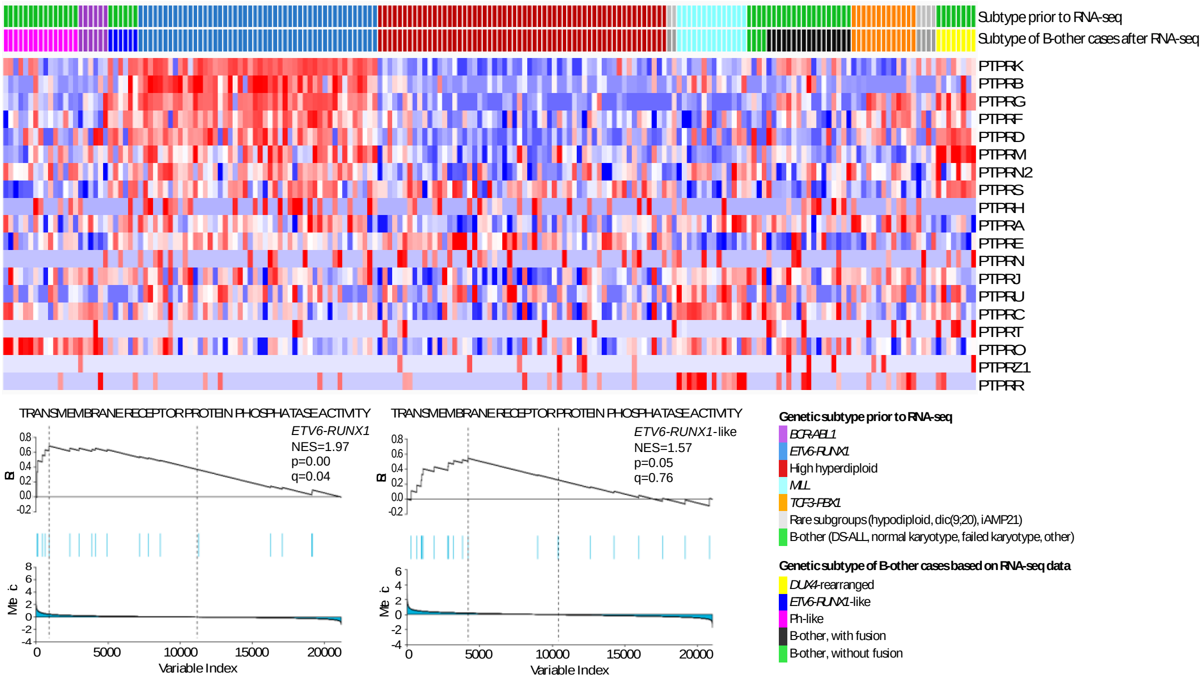

Supplementary Figure 7. Expression of transmembrane receptor protein phosphatase activity genes in 195 BCP ALLs.

Expression profile of genes involved in transmembrane receptor protein phosphatase activity, highlighted by gene set enrichment analysis to be enriched in *ETV6-RUNX1*-positive cases.

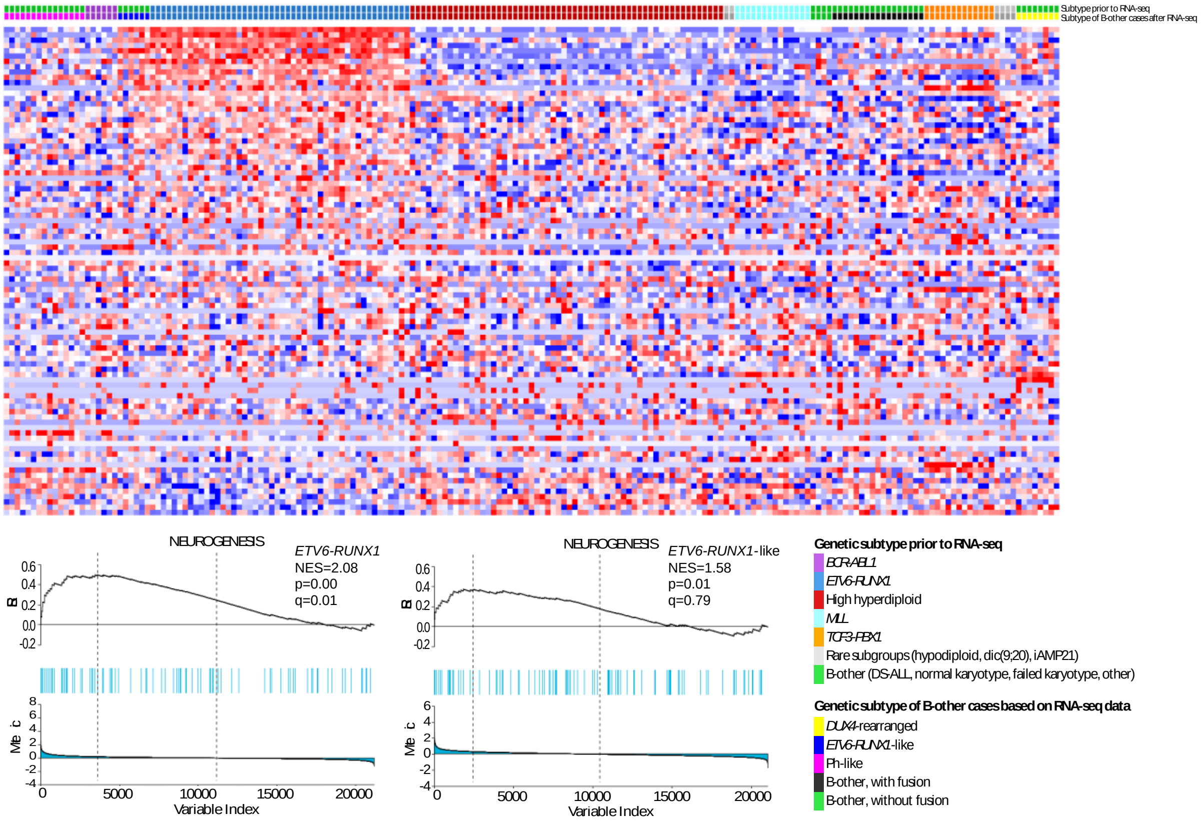

Supplementary Figure 8. Expression of neurogenesis genes in 195 BCP ALLs. Expression profile of genes involved in neurogenesis, highlighted by gene set enrichment analysis to be enriched in *ETV6-RUNX1*-positive cases.

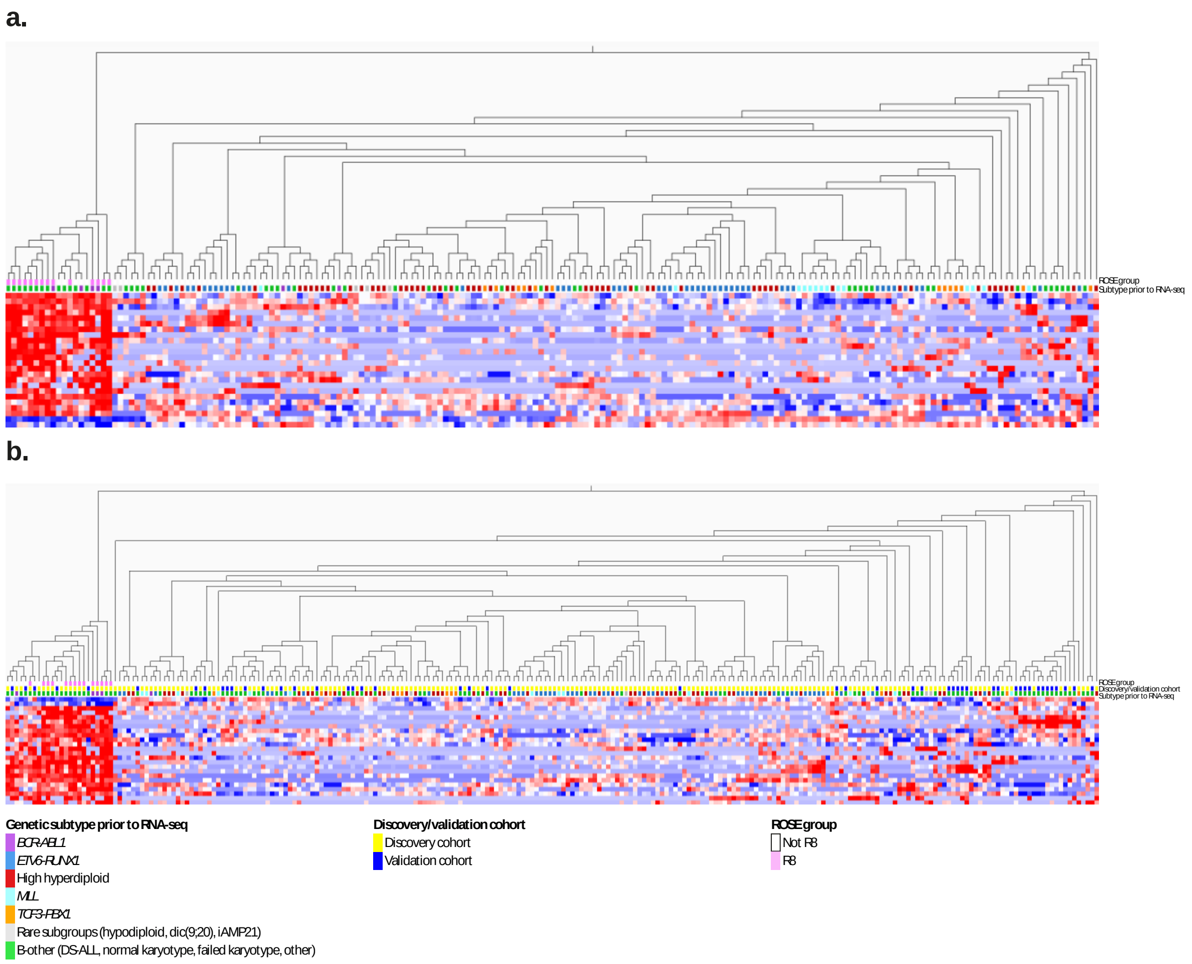

Supplementary Figure 9. Identification of cases with Ph-like gene expression. (**a**) Hierarchical clustering of 195 BCP ALL cases from the discovery cohort based on genes with a significantly (*P*<0.00001, two-sided t-test) altered expression in the 11 B-other and three *BCR-ABL1*-positive cases from the R8-cluster (**Supplementary Fig 4a**). This analysis revealed five additional cases (four B-other and one *BCR-ABL1*-positive) with a similar gene expression profile. The 15 B-other cases in this cluster were labeled "Ph-like". (**b**) Hierarchical clustering of 244 BCP ALL cases from the combined discovery and validation cohorts based on the same genes as in (a). Five cases in the validation cohort cluster with the Ph-like cases. Of these, one case (case val_25) exhibited an *ETV6-RUNX1*-like gene expression profile and harbored an out of frame *ETV6-BCL2L14* gene fusion. The remaining four cases were labeled "Ph-like".

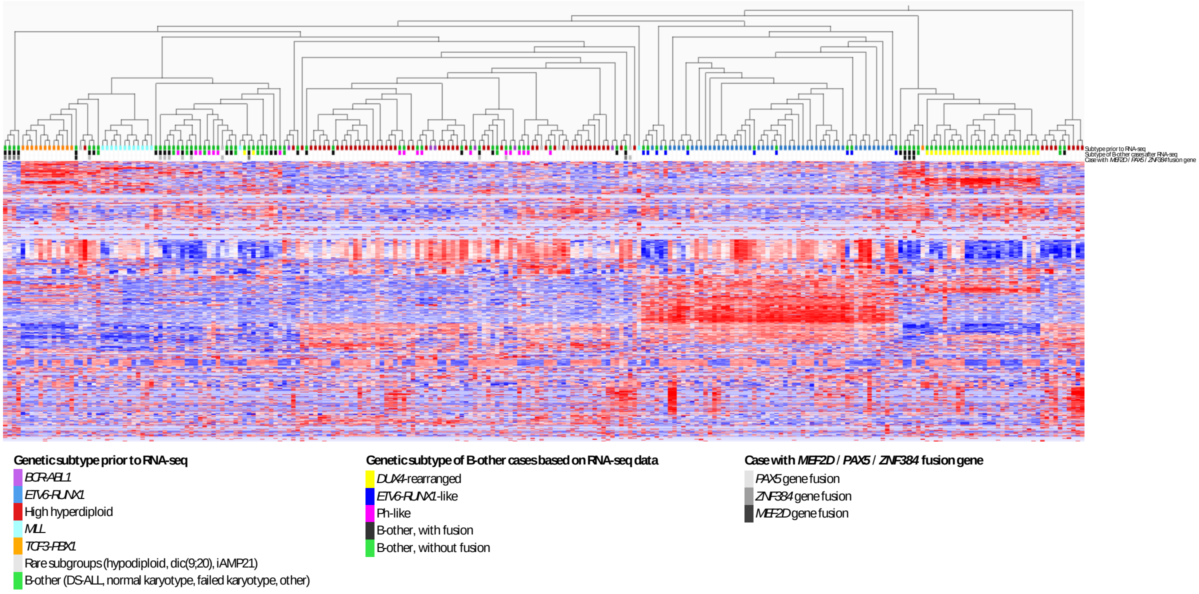

Supplementary Figure 10. Unsupervised hierarchical clustering analysis of 244 BCP ALL cases in the combined discovery and validation cohorts.
The clustering is based on 631 genes that were retained after the variance threshold was set to 0.285. A total of 10 *ETV6-RUNX1*-negative cases cluster together with the *ETV6-RUNX1*-positive cases, 6 from the discovery cohort and 4 from the validation cohort.

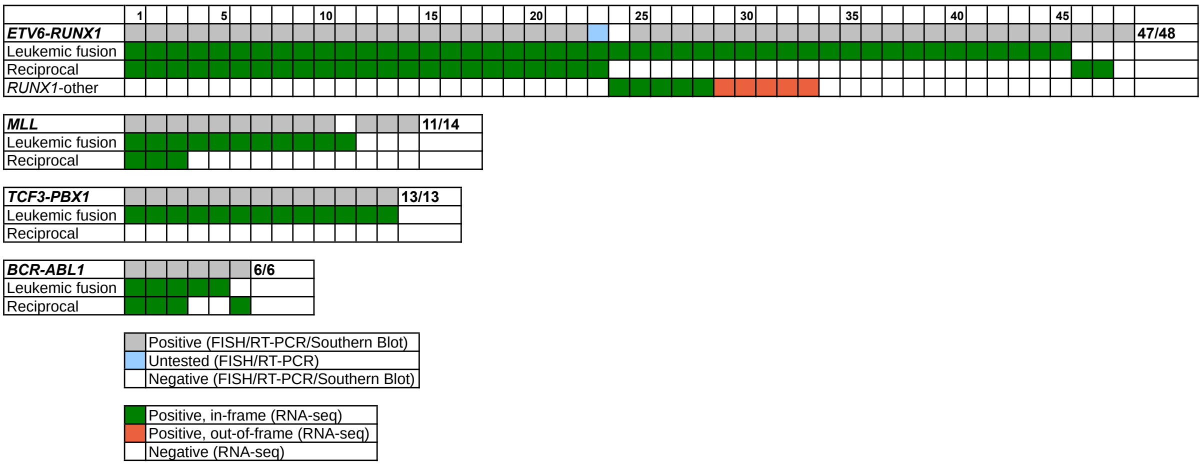

Supplementary Figure 11. Concordance between fusion gene detection by RNA-seq and directed methods.

Concordance between fusion gene detection by RNA-seq and directed methods (RT-PCR, FISH, or Southern blot). *ETV6-RUNX1* or *RUNX1-ETV6* fusions could be detected by RNA-seq in 45/46 cases that had been positive by RT-PCR or FISH. In addition, *ETV6-RUNX1* fusions were detected in one case that had not been tested and in one case that had been negative by RT-PCR. The latter fusion had a rare breakpoint that was not detectable by the RT-PCR assay. *MLL* fusions were detected by RNA-seq in 10/13 cases that had been positive by FISH, RT-PCR, or Southern blot. In addition, one case that had been negative for MLL-rearrangements by FISH harbored an intrachromosomal *MLL-USP2* fusion. *TCF3-PBX1* could be detected by RNA-seq in all 13 cases where the fusion had been detected by RT-PCR. *BCR-ABL1* or *ABL1-BCR* fusions could be detected in all cases that had been positive for *BCR-ABL1* by RT-PCR.

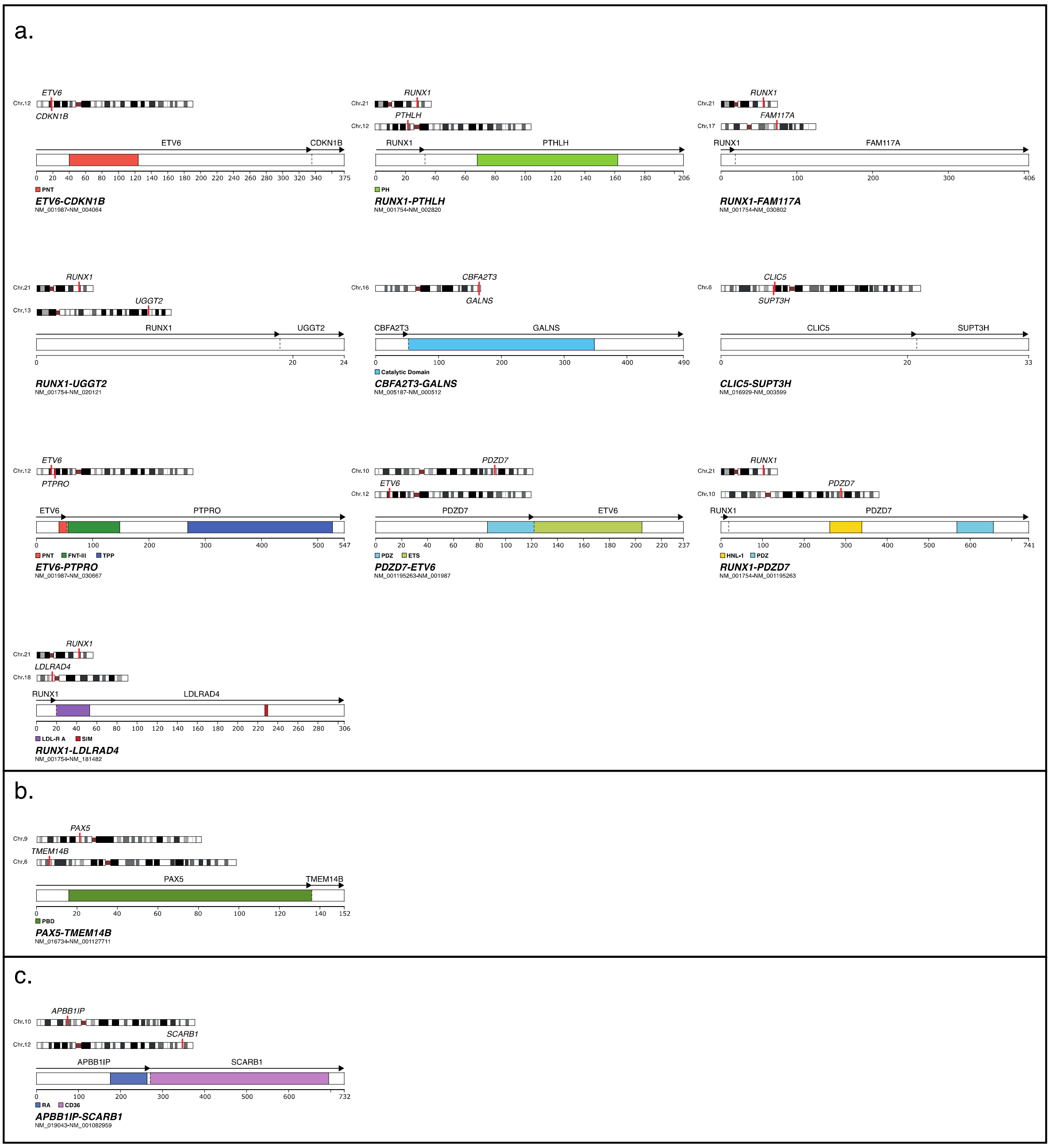

Supplementary Figure 12. Gene positions and protein domains for the 25 novel fusion genes.

Gene positions and protein domains for the 25 novel fusion genes, divided by subtype: (**a**) *ETV6-RUNX1*-positive, (**b**) High hyperdiploid, (**c**) iAMP21, (**d**) Ph-like, (**e**) *ETV6-RUNX1*-like, and (**f**) B-other, with fusion.

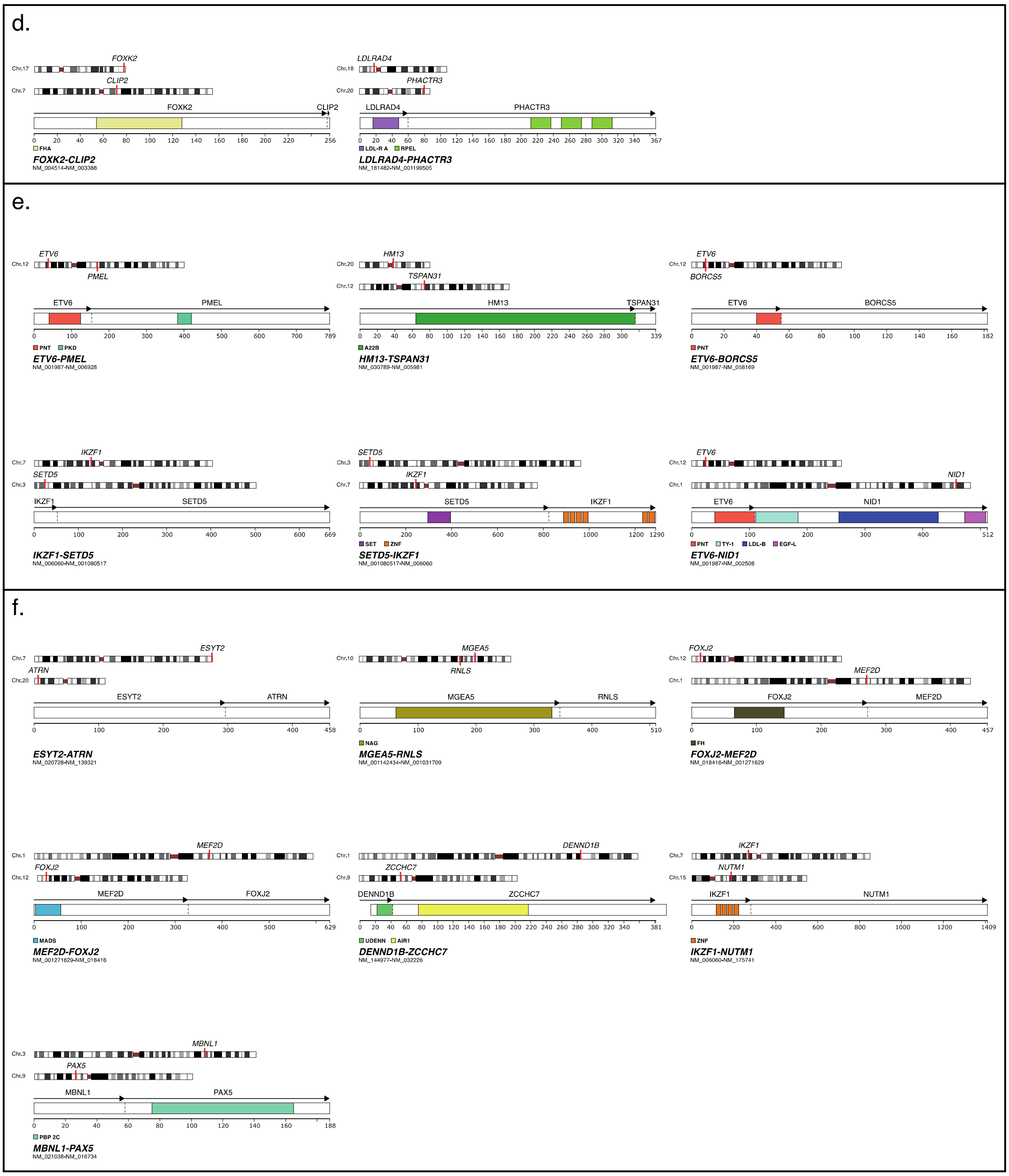

d.
FOXK2-CLIP2
NM_004514-NM_003388
LDLRAD4-PHACTR3
NM_181482-NM_001199505
e.
ETV6-PMEL
NM_001987-NM_006928
HM13-TSPAN31
NM_030789-NM_005981
ETV6-BORCS5
NM_001987-NM_058169
IKZF1-SETD5
NM_006060-NM_001080517
SETD5-IKZF1
NM_001080517-NM_006060
ETV6-NID1
NM_001987-NM_002508
f.
ESYT2-ATRN
NM_020728-NM_139321
MGEA5-RNLS
NM_001142434-NM_001031709
FOXJ2-MEF2D
NM_018416-NM_001271629
MEF2D-FOXJ2
NM_001271629-NM_018416
DENND1B-ZCCHC7
NM_144977-NM_032226
IKZF1-NUTM1
NM_006060-NM_175741
MBNL1-PAX5
NM_021038-NM_016734

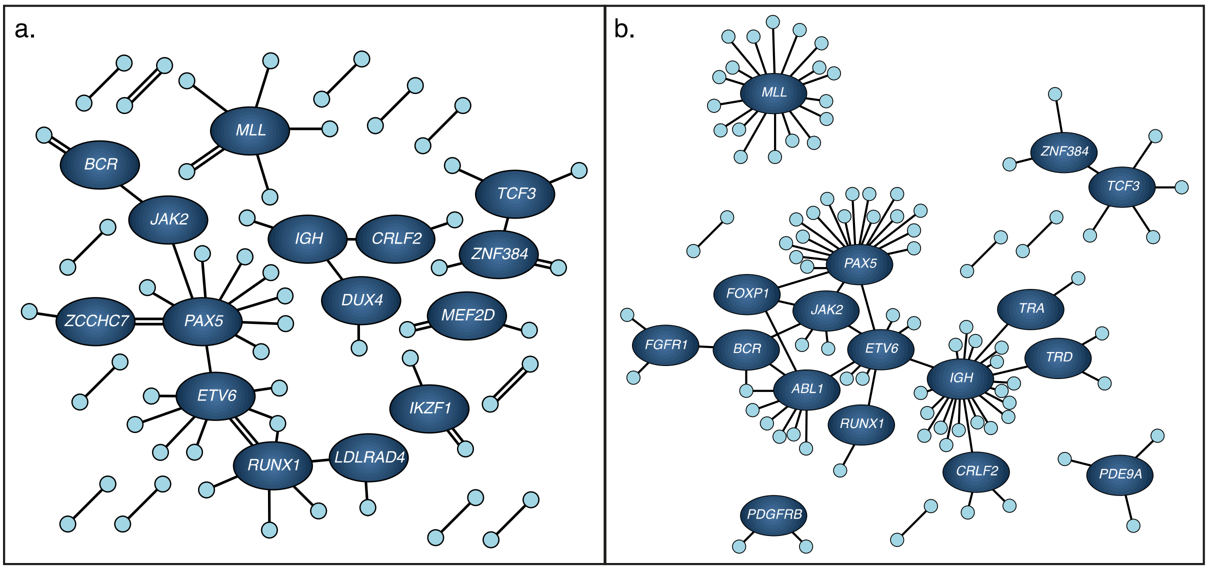

Supplementary Figure 13. Gene fusion network analysis.
Gene fusion network analysis of (**a**) in-frame fusions detected in 244 BCP-ALL cases in the combined discovery and validation cohorts, and (**b**) fusions in BCP-ALL cases from literature data[3]. Genes with two or more fusion partners (from different cases) are indicated by their gene symbol. Only gene fusions discovered using guided methods (i.e. not deep sequencing) were included in the literature data.

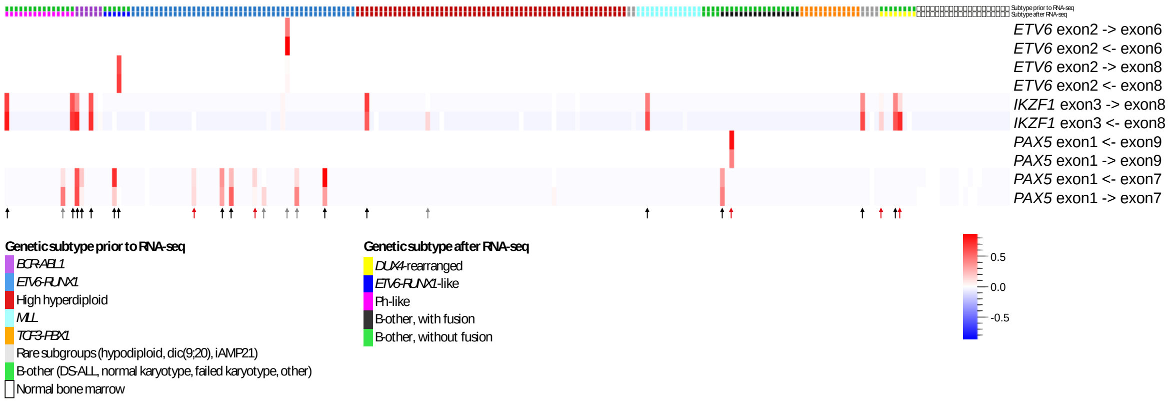

Supplementary Figure 14. Leukemia specific splice variants in *ETV6*, *PAX5*, and *IKZF1.*
Heatmap illustrating the presence of leukemia specific splice variants in *ETV6*, *PAX5*, and *IKZF1.* All splice junctions not present in a reference transcript and not detected in 20 sorted bone marrow populations (from four individual donors) are depicted. *CDKN2A* was also analyzed, but no leukemia specific junctions were identified. Leukemia specific junctions in *ETV6*, *PAX5*, and *IKZF1* with a relative fraction above 0.1 was present in 25 cases, indicated by arrows. Black arrows indicate cases with splice junctions concordant with deletions detected by SNP array, red indicates discordance with SNP array data, and grey indicates cases with no SNP array data available.

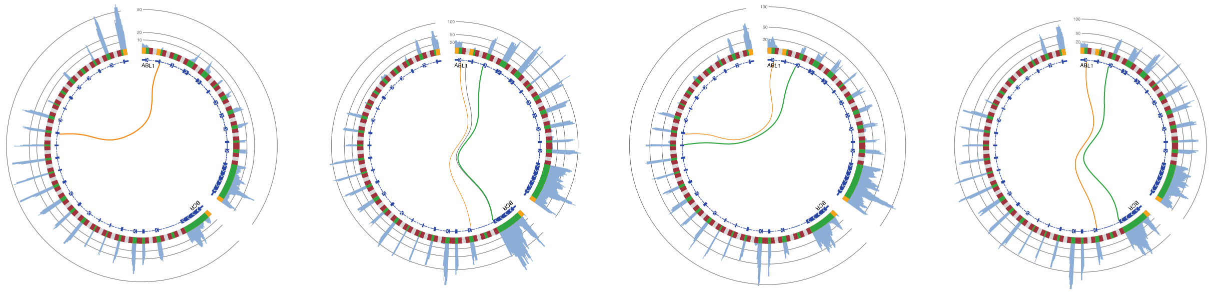

Case 51 Case 139 Case 154 Case 169

Supplementary Figure 15. Fusion junctions detected in 4 *BCR-ABL1*-positive cases. Genes are arranged clockwise by genomic position. The outer circle represents the genomic region encompassing the indicated genes. Yellow indicates untranslated regions, green indicates coding exons, and red and grey indicate intronic regions (the latter are not to scale). The inner circle represents one or two overlaid reference transcripts of the indicated gene. Coding exons are indicated by a thick line with white arrows indicating the direction of the gene, introns are indicated by a thin or dashed line, and untranslated regions are indicated by a medium thick line. Connecting lines between transcripts illustrate fusion breakpoints detected by at least three reads. Green lines indicate fusions breakpoints for *BCR-ABL1* and orange lines indicate fusion breakpoints for *ABL1-BCR*, as inferred from the position of the breakpoints.

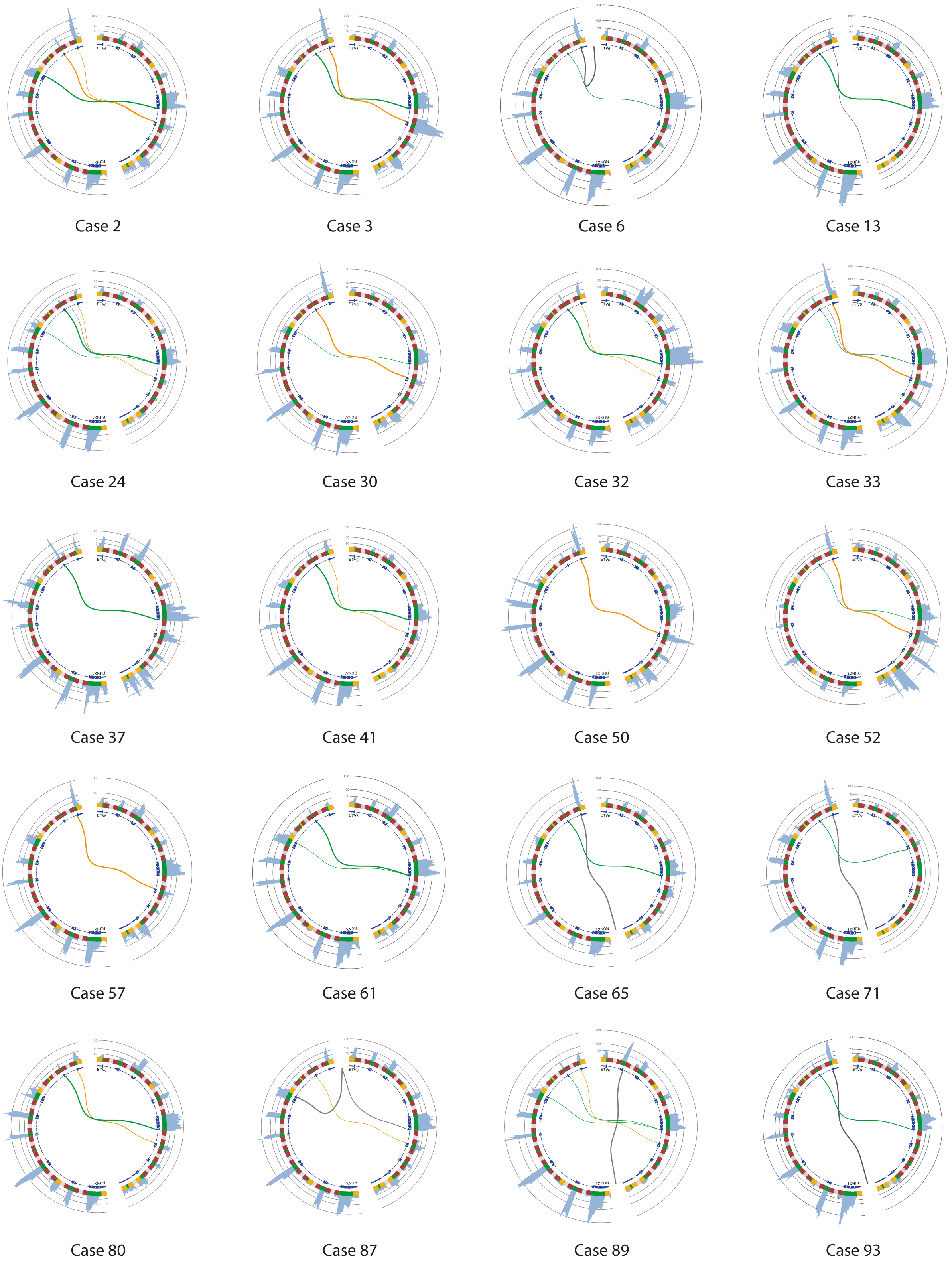

Supplementary Figure 16. Fusion junctions detected in 44 *ETV6-RUNX1*-positive cases.

Connecting lines between transcripts illustrate fusion breakpoints detected by at least three reads. Green lines indicate fusions breakpoints for *ETV6-RUNX1* and orange lines indicate fusion breakpoints for *RUNX1-ETV6*, as inferred from the position of the breakpoints. Grey lines indicate fusion breakpoints involving an intron or a gene not depicted, regardless of the inferred fusion direction.

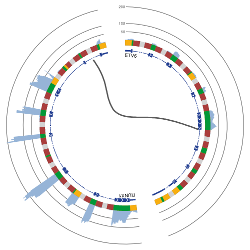

Case 99

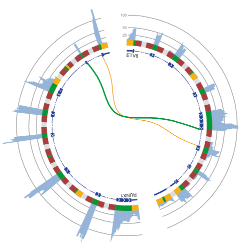

Case 114

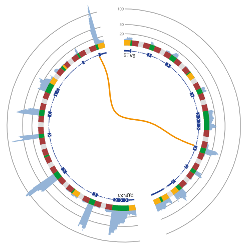

Case 117

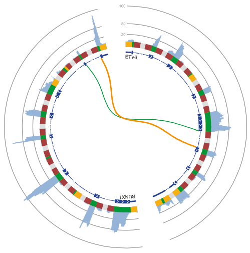

Case 118

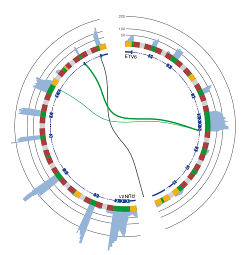

Case 123

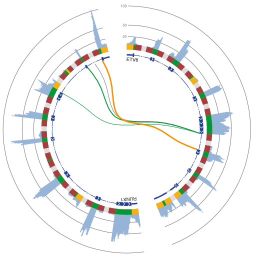

Case 126

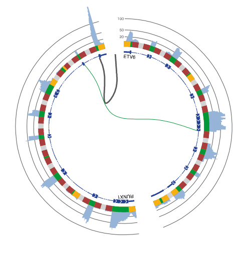

Case 129

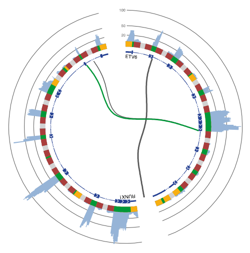

Case 131

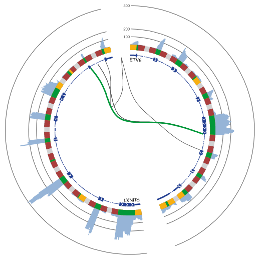

Case 132

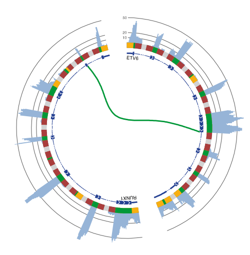

Case 134

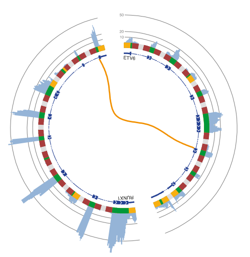

Case 138

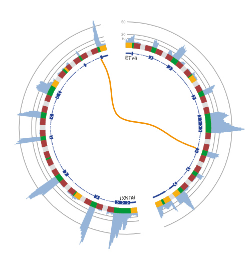

Case 142

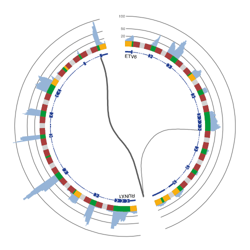

Case 143

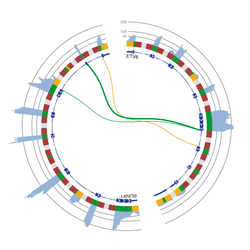

Case 149

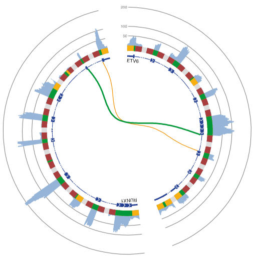

Case 155

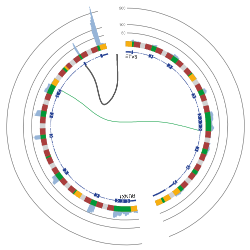

Case 168

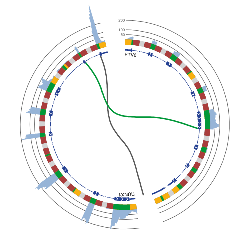

Case 171

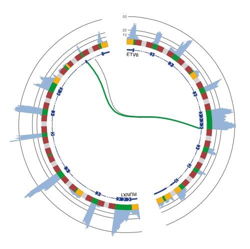

Case 178

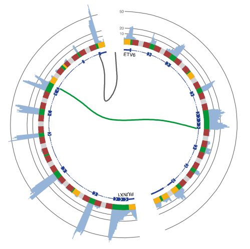

Case 180

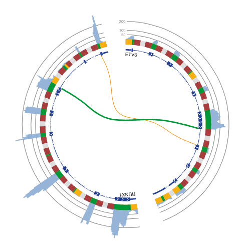

Case 186

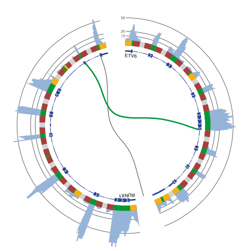

Case 187

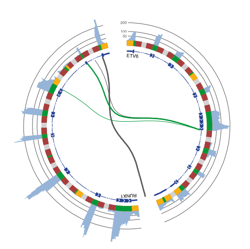

Case 189

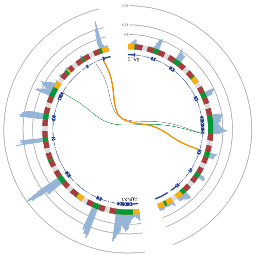

Case 190

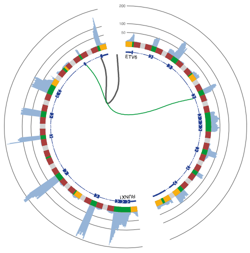

Case 193

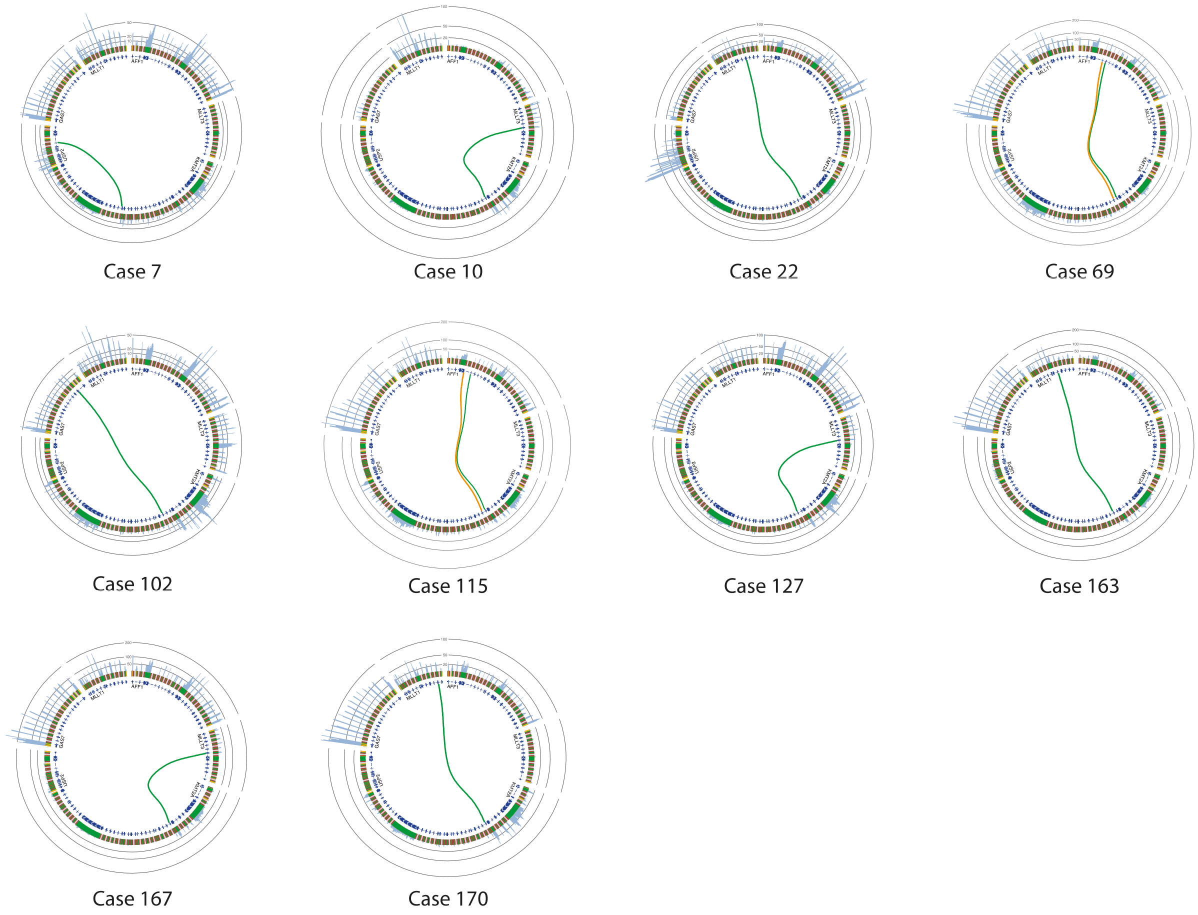

Supplementary Figure 17. Fusion junctions detected in 10 cases positive for *MLL*-fusions.

Connecting lines between transcripts illustrate fusion breakpoints detected by at least three reads. Green lines indicate breakpoints where *MLL* is the 5' partner and orange lines indicate fusion breakpoints where *MLL* is the 3' partner, as inferred from the position of the breakpoints.

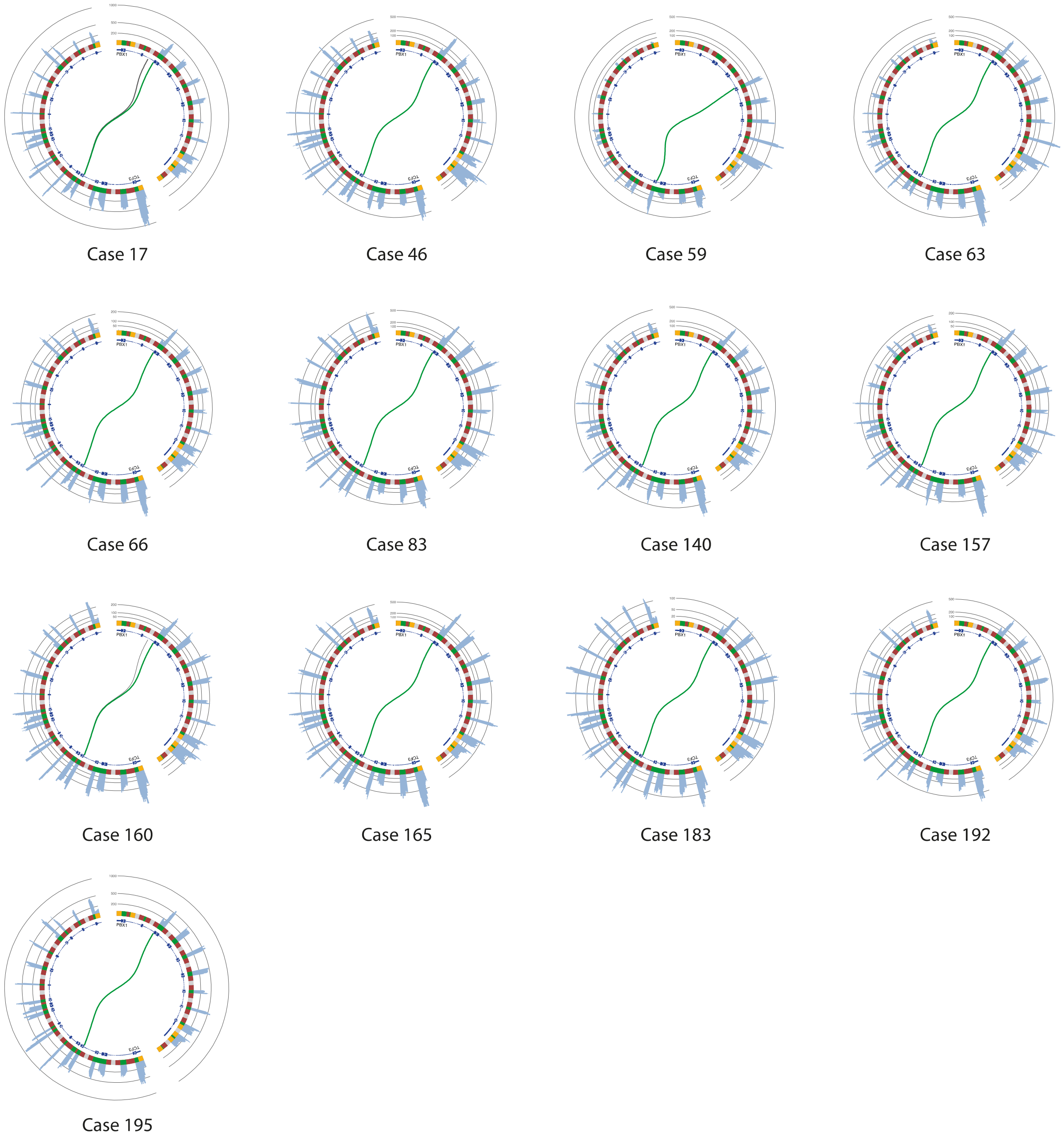

Supplementary Figure 18. Fusion junctions detected in 13 *TCF3-PBX1*-positive cases.

Connecting lines between transcripts illustrate fusion breakpoints detected by at least ten reads. Green lines indicate fusions breakpoints for *TCFR3-PBX1*. Grey lines indicate fusion breakpoints involving an intron.

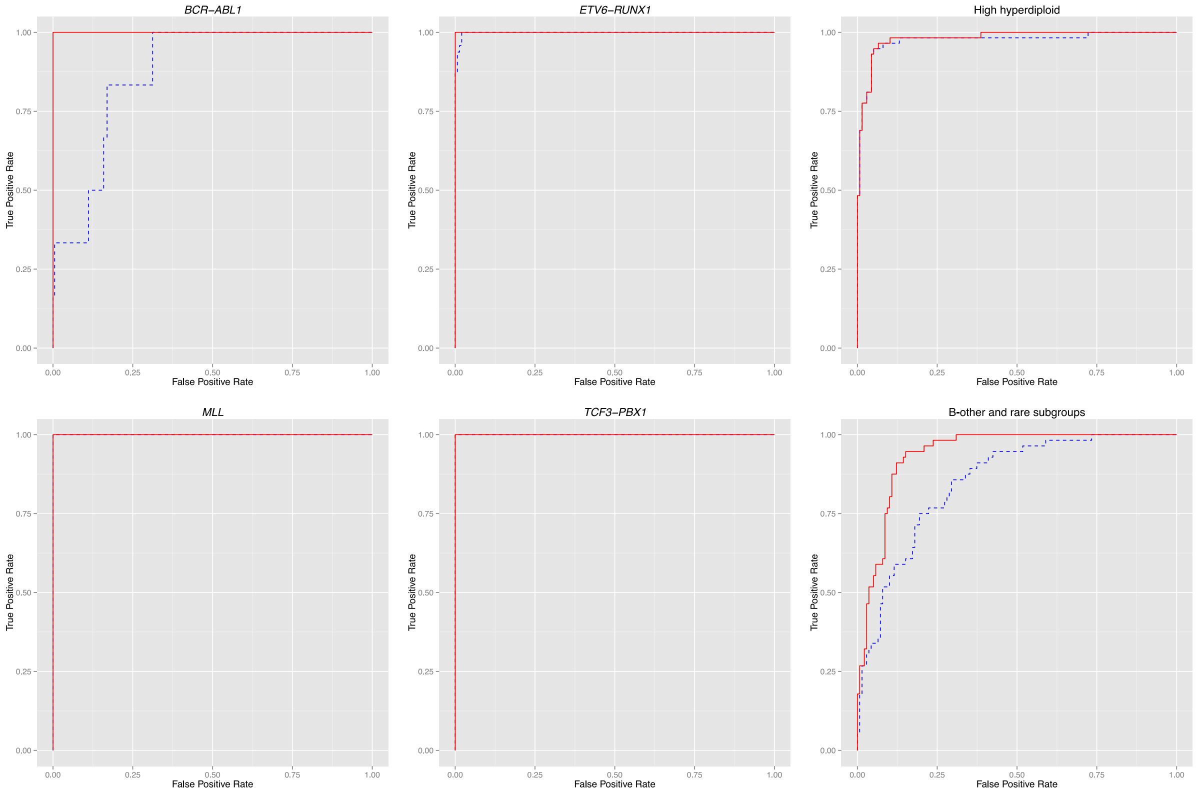

Supplementary Figure 19. Classification of 195 BCP ALL cases from the discovery cohort based on gene expression data and gene fusion data.

True positive rates versus false positive rates plotted for classifiers based on 1) both gene fusion and gene expression data (red) and 2) only gene expression data (dashed blue). In total, 180/195 samples (92%) were correctly classified when utilizing both data sets, compared to 174/195 samples (89%) when only expression data was used.

Case	Gene fusions (RNA-seq)	Deleted genes (SNP-array)	Targeted *ETV6-RUNX1* analysis		Karyotype
			FISH	RT-PCR	
**Case 64**	*ETV6-PMEL*<br>*HM13-TSPAN31*<br>*IKZF1-CDK2*	Not done	Negative for *ETV6-RUNX1* (wcp12)	Negative	46,XY,del(1)(q21),-7,-12,+mar,inc
**Case 68**	*ETV6-LOH12CR1*	*ETV6, IKZF1, PAX5*	Negative for *ETV6-RUNX1*	Negative	46,XX
**Case 85**	*P2RY8-CRLF2*<br>*IKZF1-SETD5*<br>*SETD5-IKZF1*	*ETV6, BTG1*	Not done	Negative	47,XY,t(3;7)(p25;p12),+21c,inc
**Case 105**	None	Not done	Not done	Negative	46,XY
**Case 111**	None	*ETV6, IKZF1*		Negative	45,XX,dic(7;12)(p11;p11)
**Case 176**	*ETV6-NID1*	*ETV6, IKZF1*		Negative	45,XY,?der(1)t(1;12)(q44;p13),dic(7;12)(p11;p11),del(12)(p13),-14,ins(14;?)(q24;?),+der(?)t(?;12)(?;p13) (partly based on FISH)

Supplementary Table 1. Summary of genetic aberrations detected in six BCP ALL cases with *ETV6-RUNX1*-like gene expression profile.

Analysis	Primer sequence
ERG deletion	Forward: 5'CTC CTC CAG CGA CTA TGG AC 3'<br>Reverse: 5'GCG GCT GAG CTT ATC GTA GT 3'
FLT3 internal tandem duplication	Forward: 5´GCA ATT TAG GTA TGA AAG CCA GC 3´<br>Reverse: 5´CTT TCA GCA TTT TGA CGG CAA CC 3´
FLT3 activating mutations	Forward: 5´ATC ATC ATG GCC GCT CAC 3´<br>Reverse: 5´ GCA CTC AAA GGC CCC TAA CT 3´
NRAS exon 2 (codons 12 and 13)	Forward: 5'-GTACTGTAGATGTGGCTCGCCA-3'<br>Reverse: 5'-GCCTCACCTCTATGGTGGGAT-3'
NRAS exon 3 (codon 61)	Forward: 5'-ACCCCCAGGATTCTTACAGAA-3'<br>Reverse: 5'-GCCTGTCCTCATGTATTGGTCT-3'
KRAS exon 2 (codons 12 and 13)	Forward: 5'-TGTATTAACCTTATGTGTGACATGTTC-3'<br>Reverse: 5'-CACCAGTAATATGCATATTAAAACAAG-3'
KRAS exon 3 (codon 61)	Forward: 5'-CTGTGTTTCTCCCTTCTCAGGATTC-3'<br>Reverse: 5'-AAGAAAGCCCTCCCCAGTCCT-3'
PTPN11 exon 3	Forward: 5'-CCGACGTGGAAGATGAGATCTG-3'<br>Reverse: 5'-CATACACAGACCGTCATGCATTTC-3'
PTPN11 exon 13	Forward: 5'-CTCTGAGTCCACTAAAAGTTGTGCAT-3'<br>Reverse: 5'-AGCAAGAGAATGAGAATCCGCA-3'

Supplementary Table 2. Primers used for detecting somatic mutations.

Supplementary references

1. Thorvaldsdóttir, H., Robinson, J. T. & Mesirov, J. P. Integrative Genomics Viewer (IGV): high-performance genomics data visualization and exploration. *Brief. Bioinform.* **14,** 178–192 (2013).
2. Harvey, R. C. *et al.* Identification of novel cluster groups in pediatric high-risk B-precursor acute lymphoblastic leukemia with gene expression profiling: correlation with genome-wide DNA copy number alterations, clinical characteristics, and outcome. *Blood* **116,** 4874–4884 (2010).
3. Mitelman, F., Johansson, B. & Mertens, F. Mitelman database of chromosome aberrations and gene fusions in cancer. (2016). at <http://cgap.nci.nih.gov/Chromosomes/Mitelman>
